# Open-source Software Sustainability Models: Initial White Paper From the Informatics Technology for Cancer Research Sustainability and Industry Partnership Working Group

**DOI:** 10.2196/20028

**Published:** 2021-12-02

**Authors:** Ye Ye, Seemran Barapatre, Michael K Davis, Keith O Elliston, Christos Davatzikos, Andrey Fedorov, Jean-Christophe Fillion-Robin, Ian Foster, John R Gilbertson, Andras Lasso, James V Miller, Martin Morgan, Steve Pieper, Brigitte E Raumann, Brion D Sarachan, Guergana Savova, Jonathan C Silverstein, Donald P Taylor, Joyce B Zelnis, Guo-Qiang Zhang, Jamie Cuticchia, Michael J Becich

**Affiliations:** 1 Department of Biomedical Informatics School of Medicine University of Pittsburgh Pittsburgh, PA United States; 2 Axiomedix, Inc. Bedford, MA United States; 3 PHEMI Systems Corp. Vancouver, BC Canada; 4 tranSMART foundation Wakefield, MA United States; 5 Department of Radiology School of Medicine University of Pennsylvania Philadelphia, PA United States; 6 Brigham and Women's Hospital Harvard Medical School Boston, MA United States; 7 Kitware Inc. Clifton Park, NY United States; 8 Department of Computer Science University of Chicago Chicago, IL United States; 9 The Perk Lab for Percutaneous Surgery School of Computing Queen's University Kingston, ON Canada; 10 GE Global Research Niskayuna, NY United States; 11 Department of Biostatistics and Bioinformatics Roswell Park Comprehensive Cancer Center Buffalo, NY United States; 12 Isomics Inc. Cambridge, MA United States; 13 Globus University of Chicago Chicago, IL United States; 14 Boston Children’s Hospital Harvard Medical School Boston, MA United States; 15 The University of Texas Health Science Center at Houston Houston, TX United States

**Keywords:** open-source software, sustainability, licensing model, financial model, product management, cancer informatics

## Abstract

**Background:**

The National Cancer Institute Informatics Technology for Cancer Research (ITCR) program provides a series of funding mechanisms to create an ecosystem of open-source software (OSS) that serves the needs of cancer research. As the ITCR ecosystem substantially grows, it faces the challenge of the long-term sustainability of the software being developed by ITCR grantees. To address this challenge, the ITCR sustainability and industry partnership working group (SIP-WG) was convened in 2019.

**Objective:**

The charter of the SIP-WG is to investigate options to enhance the long-term sustainability of the OSS being developed by ITCR, in part by developing a collection of business model archetypes that can serve as sustainability plans for ITCR OSS development initiatives. The working group assembled models from the ITCR program, from other studies, and from the engagement of its extensive network of relationships with other organizations (eg, Chan Zuckerberg Initiative, Open Source Initiative, and Software Sustainability Institute) in support of this objective.

**Methods:**

This paper reviews the existing sustainability models and describes 10 OSS use cases disseminated by the SIP-WG and others, including 3D Slicer, Bioconductor, Cytoscape, Globus, i2b2 (Informatics for Integrating Biology and the Bedside) and tranSMART, Insight Toolkit, Linux, Observational Health Data Sciences and Informatics tools, R, and REDCap (Research Electronic Data Capture), in 10 sustainability aspects: governance, documentation, code quality, support, ecosystem collaboration, security, legal, finance, marketing, and dependency hygiene.

**Results:**

Information available to the public reveals that all 10 OSS have effective governance, comprehensive documentation, high code quality, reliable dependency hygiene, strong user and developer support, and active marketing. These OSS include a variety of licensing models (eg, general public license version 2, general public license version 3, Berkeley Software Distribution, and Apache 3) and financial models (eg, federal research funding, industry and membership support, and commercial support). However, detailed information on ecosystem collaboration and security is not publicly provided by most OSS.

**Conclusions:**

We recommend 6 essential attributes for research software: alignment with unmet scientific needs, a dedicated development team, a vibrant user community, a feasible licensing model, a sustainable financial model, and effective product management. We also stress important actions to be considered in future ITCR activities that involve the discussion of the sustainability and licensing models for ITCR OSS, the establishment of a central library, the allocation of consulting resources to code quality control, ecosystem collaboration, security, and dependency hygiene.

## Introduction

### Background

The Informatics Technology for Cancer Research (ITCR) program [1] was established by the National Cancer Institute (NCI) in 2012 to create an ecosystem of open-source software (OSS) that serves the needs of cancer research. ITCR supports informatics technology development initiated by cancer research investigators and includes 4 extramural divisions: cancer biology, cancer control and population science, cancer prevention, and cancer treatment and diagnosis. The coordinating body for ITCR is the NCI Center for Biomedical Informatics and Informatics Technology.

The specific goals of ITCR include (1) promoting the integration of informatics technology development with hypothesis-driven cancer research and translational or clinical investigations; (2) providing flexible, scalable, and sustainable support using multiple mechanisms matched to the various needs and different stages of informatics technology development throughout the development life cycle; (3) promoting interdisciplinary collaboration and public–private partnerships in technology development and distribution; (4) promoting data sharing and development of informatics tools to enable data sharing; (5) promoting technology dissemination and software reuse; (6) promoting communication and interaction among development teams; and (7) leveraging the NCI program expertise and resources across the institute and bridging gaps in the existing NCI grant portfolios for informatics.

The scope of the ITCR program is to serve informatics needs that span the cancer research continuum. The ITCR program provides a series of funding mechanisms that support informatics resources across the development life cycle, including the creation of innovative methods and algorithms (R21), early-stage software development (R21), advanced stage software development (U24), and the sustainment of high-value resources (U24) on which the cancer research and translational informatics community has come to depend (Table 1). The program also offers supplements (*competitive revisions*) to currently funded NCI grantees to incorporate ITCR technologies into their ongoing research. Current funding opportunities are available on the ITCR website [2].

**Table 1:** Informatics Technology for Cancer Research (ITCR) funding mechanisms.

Mechanism	Purpose	Awards before September 9, 2020	Direct cost cap
R21	Innovative informatics methods and algorithms	25	US $275,000 over 2 years
U01	Early-stage software development	34	US $300,000 per year for up to 3 years
U24	Advanced stage software development	40	US $600,000 per year for up to 5 years
U24	Sustainment of high-value resources	6	No budget cap and up to 5 years of support
Competitive revisions (new)	Adoption, adaptation, and integration of ITCR tools and resources	1	US $100,000 per year for up to 2 years

**Figure 1:**
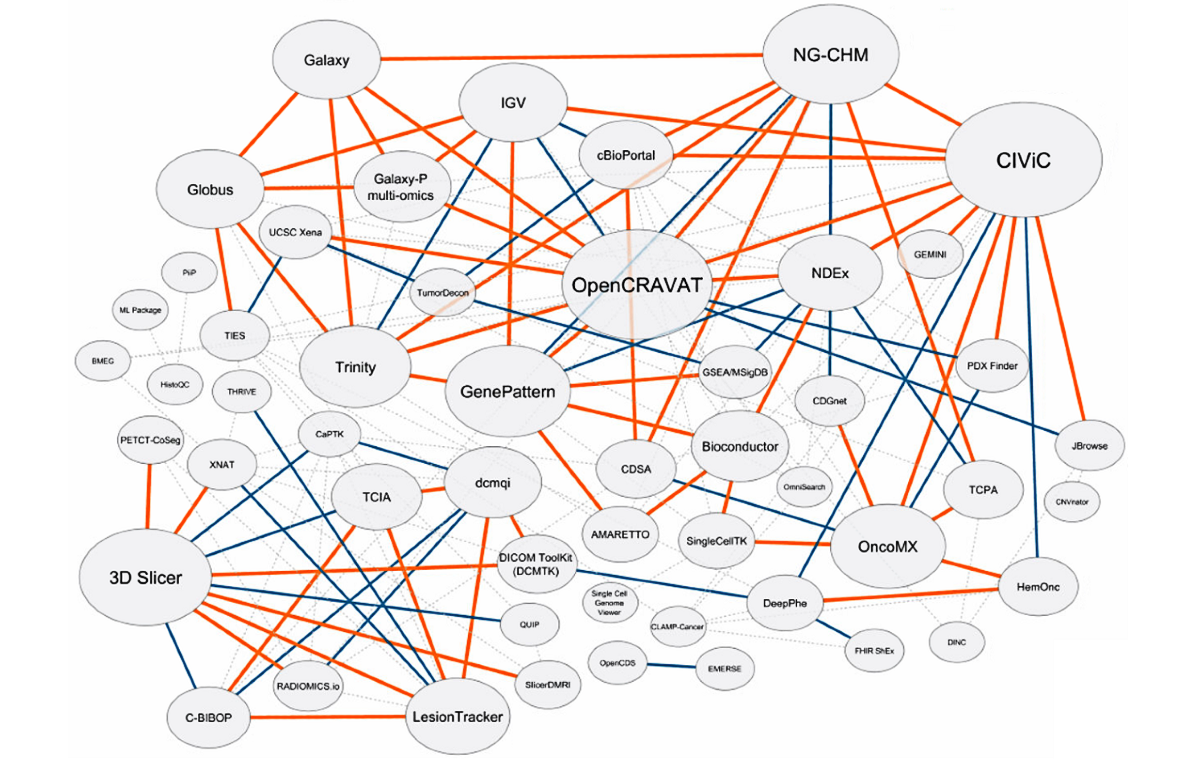
The map of Informatics Technology for Cancer Research projects.

This series of funding mechanisms is innovative and unique across all National Institutes of Health (NIH) institutes and centers. These mechanisms address a fundamental need to create a computational infrastructure that is interoperable and collaborative, linking many informatics and computational biology teams performing translational informatics. The ITCR ecosystem has grown substantially and now includes 55 funded efforts that are highly collaborative, as evidenced by its *connectivity map* (Figure 1). This map is copied from the Network Data Exchange website [3,4]. In this map, each node represents a project funded under ITCR. Links among these nodes represent connections among projects. Existing connections are represented by orange solid lines, ongoing connections by blue solid lines, and proposed connections by gray dashed lines. The node size is determined by the connectivity score, which is calculated by assigning 0 points for each proposed connection, 1 point for each ongoing connection, and 3 points for each existing connection. A large node usually indicates that the project has many existing connections with other projects. The connectivity scores are available on the Network Data Exchange website.

As the ITCR program moves into its second phase, it faces the challenge of long-term sustainability for the software being developed by its grantees. Whether viewed from the angle of a single funded project or all ITCR-funded projects, some of the software will naturally *graduate* upon reaching maturity to leave room for continuing innovation through the program. As mature projects often lead to complex and successful products based on years of investment in human effort, funding, and cumulative expertise, these projects need to move into the next phase of support rather than risk being abandoned.

Addressing the challenge of the projects’ long-term sustainability was the primary task of the ITCR sustainability and industry partnership working group (SIP-WG) [5], which was convened in 2019. The working group initially set the goals of addressing 4 topics of interest to the translational cancer informatics community: (1) to publish a collection of case studies of successfully disseminated software products supported by open-source licenses and to provide practical examples of approaches that have proven viable for licensing and sustainability, (2) to develop a workflow or decision tree to support informed decision-making consistent with ITCR expectations and the future licensing needs of open-source tools, (3) to provide a licensing consultancy service in collaboration with the ITCR program, and (4) to develop a collection of business model archetypes that can serve as starting templates and to formally document the dissemination and sustainability plans for new software development initiatives. The ITCR licensing resources will represent *best practice* approaches and leverage our extensive network of relationships with organizations such as the Open Source Initiative, the Software Sustainability Institute, and the Chan Zuckerberg Initiative to maintain relevant knowledge in this field. As described above, the first major topic—publication of case studies—is the subject of this paper. The remaining 3 topics will be the focus of future white papers and manuscripts by the ITCR SIP-WG.

### Literature Review

We briefly introduce several software sustainability models that are present in the literature [6-8]. First, Aartsen et al [8] described 2 models for sustaining digital assets from public–private partnerships in medical research: the not-for-profit organization model and the distributed network model. The *not-for-profit organization model* uses, for example, a foundation (also discussed by Kuchinke et al [6]) as the backbone organization to assure the maximum value of the assets. The Apache Software Foundation is one such example. An advantage of nonprofits is that they can take a long-term view. The sustainability of nonprofits can be mitigated through memberships. The concept of a foundation has the advantage that the development of an artifact is strongly influenced by academic users, so its design can be focused on scientific goals instead of commercial ones. The disadvantage of the not-for-profit organization model is its dependency on one organization for all digital assets. The *distributed network model* is built on the premise that individual partners who contribute to the development of digital assets have a stake in seeing these assets sustained and gaining future value through further development. The disadvantage of the distributed network model lies in the conflicting missions of research and industry; organizations with a research mission do not focus on producing digital assets that are ready to be commercialized by industry.

Gabella et al [9] provided a comprehensive review that adds 10 models for the sustainability of assets, including 4 noncommercial and 6 commercial models. As a noncommercial model, the *national funding model* supports infrastructure directly through noncyclical funding programs. On the other hand, in the *infrastructure model*, funding agencies set aside a fixed percentage of their research grant volumes to be redistributed among core data resources according to well-defined selection criteria. In the *institutional support model*, funds are provided internally from the institution, whereas the *donation model* depends on external philanthropic funding. In terms of the 6 commercial models, the *content licensing or industrial support model* requires commercial users to pay a fee for access and for-profit use, with the assets being free for noncommercial users (also discussed by Kuchinke et al [6]). The *user subscription model* (also discussed by Chang et al [7]) relies on a subscription for a set period. The *freemium model* (also discussed in Chang et al [7]) provides a core that is free, with add-ons requiring a fee. The *razor and blades model* (also discussed in a Wikipedia introduction for business models of OSS [10] as a commercial model) offers a free initial trial (*razor*) that encourages the continuing future purchases of follow-up services (*blades*). The *mixed model* relies on multiple diversified funding streams. For instance, a common mixed model practice is the combination of OSS with services (provided by companies) on installation, configuration, and troubleshooting. Linux is a familiar example of this. However, the Linux model relies on a large user base, which may not necessarily be the case with biomedical research tools.

In addition to the models discussed in last paragraph, the *macro research and development infrastructure* is based on funding that comes from governmental research grants or from research grants from local or international partner institutes [7]. The *split licensing model* offers a free version under a general public license (GPL) and a commercial version with its own license that does not allow software redistribution (eg, MySQL [Sun Microsystems, Inc] and openClinica [OpenClinica, LLC]) [10].

The current literature has also discussed the importance of the strength and health of the community behind a software product [11-13]. Iaffaldano et al [11] used the sleep stage metaphor to describe developer cycles: the awake stage is when developers are active in the project, the sleep stage is when developers pause their package commit activity, and the dead stage is when developers abandon the project. They further explored the reasons for the stage transitions, listing both personal factors (eg, life event, financial, and change of interest) and project factors (eg, social, changes in the project, and role change) as playing a role. Atiq et al [12] suggested sponsoring of open-source projects in various ways as an increasing number of proprietary firms participate in, sponsor, and offer their developers for open-source projects. Jiménez et al [13] provided 4 recommendations for a sustainable open-source project: (1) making the source code publicly accessible from day 1, (2) making the software easy to discover by providing software metadata via a popular community registry, (3) adopting a licensing system that complies with the licenses of third-party dependencies, and (4) defining clear and transparent contributions, governance, and communication processes. Nyman et al [14,15] discussed code forking (implementing an existing code base found in a separate project) within the context of OSS. The right to fork code is built into the very definition of open source. Code forking can revive community interest in a project or provide an alternative to acquisitions, which was the case with MySQL after Oracle’s acquisition of Sun Microsystems. The MySQL code was forked under a different name, MariaDB, because of concerns regarding the governance and future openness of the MySQL code. Nyman and Lindman [14] state, “Given that forking ensures that any project can continue as long as there is sufficient community interest, we have previously described forking as the ‘invisible hand of sustainability’ in open source.” For specifically big biology, Prins et al [16] described the challenges of creating sustainable software solutions: most OSS are developed as prototype software, many OSS are not scaled to terabytes of data, and there is a lack of scientific attribution for software development.

## Methods

We conducted a survey among the members of the working group to select a collection of case examples of successfully disseminated software products. We asked each member to provide the *best 3* examples of sustainable OSS to serve as models for ITCR open-source projects. The survey was completed by 13 participants, most of whom were authors of this white paper and had years of experience developing OSS for cancer research. To profile the models of success in sustainability, 22 OSS use cases were provided by this survey, and the top 10 tools were then assigned to authors who were then asked to profile the following models: 3D Slicer [17], Bioconductor [18], Cytoscape [19], Globus [20], i2b2 (Informatics for Integrating Biology and the Bedside) [21] and tranSMART [22], Insight Toolkit (ITK) [23], Linux [24], Observational Health Data Sciences and Informatics (OHDSI) [25], R [26], and REDCap (Research Electronic Data Capture; provides a nonprofit end user license agreement but its code base is not open to individual developers) [27].

After reviewing the literature and discussing it in the ITCR working group, we determined that each OSS use case should be profiled according to recommendations by Nesbitt [28] in his paper: “What does a sustainable open source project look like?” Accordingly, each of the top 10 OSS use cases was profiled in the following aspects of sustainability: governance, documentation, code quality, support, ecosystem collaboration, security, legal issues, financing, marketing, and dependency hygiene. Profiling mainly relies on information that is publicly available. As some of the coauthors are key developers of 3D Slicer (AF, SP, JCFR, JVM, and AL) and Globus (IF and BER), we were able to provide more firsthand information on these 2 cases.

## Results

In this section, we examine each OSS use case in terms of these 10 sustainability aspects. Full descriptions of the OSS use cases are available in Multimedia Appendix 1.

### Governance

All 10 OSS use cases have a management committee and a technology development team. ITK and REDCap have established consortiums. The 3 models (i2b2 tranSMART, R, and Linux) have established foundations. Stakeholders usually choose a consortium management model during the early stages of software development. In a consortium model, members have stronger control over the direction of development. A consortium management model may later migrate into a foundation model. In a foundation model, the organization considers the interests of all stakeholders, encouraging more new contributors and users to participate in the software development testing process. As a result, foundations usually require serious community efforts and diverse skills (eg, fundraising) [29].

The 6 OSS tools have provided their roadmaps publicly. The i2b2 tranSMART Foundation [30] defines a road map guiding the integration of tranSMART with i2b2 [31]. The 3D Slicer’s road map [32] lists community suggestions related to a transition plan for Slicer 4.10 and the proposed changes for Slicer 5.x. Cytoscape’s road map [33] shows that it is going down a number of roads simultaneously, including Cytoscape Desktop, Cytoscape Expansion to the Cloud, and Cytoscape Community Outreach. Globus’s product road map [34] has plans to provide *research information technology as a service*. ITK’s team has been continuously updating its road map [35-37] based on feedback from its community of users and developers as well as from the medical research community. OHDSI has several roadmaps, including an architecture road map [38], a road map for CDM v6.0 [39], and a road map for webAPI [40]. On the other hand, LWN.net, a computing webzine on software for Linux and other Unix-like operating systems, points out that the free software development model is resistant to central planning in general [41]. Although not always reliable, Linux’s future can be reasonably predicted by looking at its current projects.

Regular meetings allow stakeholders to make operational decisions and set development priorities. The 3D Slicer’s core developers and users meet in person twice a year, and Globus has an annual conference for its users and subscribers. The subgroups usually have more frequent regular meetings. On the other hand, the technical advisory board of Bioconductor meets monthly to develop strategies that ensure the long-term technical suitability of the core infrastructure. To reach a broader group of potential developers and users, some models (3D Slicer and i2b2 tranSMART) provide completely open communication channels, such as web-based forums and recorded webinars.

Owing to the limited amount of public information on these 10 OSS use cases, we do not know the exact size of each core development team or the individual assignments on core infrastructure. If there is a single person handling the complicated details of a critical component, an OSS project will go adrift quickly after losing that key person.

### Documentation

All 10 OSS use cases provide documentation to users in various formats, such as user guidebooks (ITK [42], Linux [43], R [44], and 3D Slicer [45]), Wiki pages (3D Slicer [46] and i2b2 [47]), tutorials (Bioconductor [48], Globus [49], Cytoscape [50], tranSMART [51], 3D Slicer [52], and OHDSI [53]), and YouTube (Google, Inc) videos (REDCap [54], 3D Slicer [55], and Cytoscape [56]).

Further documentation is provided to new developers to encourage new contributions to OSS extensions. Bioconductor offers 3 levels of documentation—workflows, package vignettes, and function manual pages [57]—to encourage users to become developers who can make their own algorithms and approaches available to others. Similarly, the Cytoscape *App Ladder* teaches essential skills in app development [58]. R provides a variety of fully developed documentation, adequately covering 2 types of development: writing R extensions and developing R itself (by providing internal structure and coding standards) [59].

### Code Quality

Releasing software without testing could be very dangerous to its reliability and reproducibility, so rigorous tests are critical for OSS. Before propagating the latest packages to user-facing repositories, Bioconductor developers conduct tests to ensure overall package integrity and integration with current versions of package dependencies. The 3D Slicer has established infrastructure to continuously run approximately 700 tests for its core application, with the test results being publicly available [60]. However, the quality control of some of the extensions of the 3D Slicer is slightly weaker than that of the core application. The extension contributors themselves manage the code quality and tests, and the 3D Slicer’s core developer team does not enforce or verify these extensions. Cytoscape developers use Jenkins to build software projects continuously and test packages thoroughly before releasing them. Globus uses a continuous integration environment, automated tests, multiple prerelease environments, and documented, standardized, human quality assurance testing to ensure code quality, with at least one engineer other than the code author reviewing the code before releasing it to production. Both i2b2 and tranSMART have extensive automated and manual testing as part of their well-defined release processes. ITK had automated nightly builds and tests as far back as 1999, being an early adopter of this software engineering best practice before the widespread adoption of continuous integration and GitHub (GitHub, Inc). R provides extensive support to facilitate external developers’ package testing and release, which includes release guidelines, software packages, and servers for testing [61]. A few models (3D Slicer [62], ITK, and R [63]) enforce a consistent coding style.

### Support

All OSS use cases provide support to users and new developers. For example, the OHDSI community provides 2 support channels: the community-based discourse forum provides support for implementing OHDSI tools, proposing or participating in network research studies, and requesting information on OHDSI-related topics [64]; and the GitHub project sites of OHDSI manage specific technical questions through tickets that anyone can issue [65]. Globus has several support options: web-based self-help tools, listserv groups, and a ticketing submission system with a responsive support team. R mainly relies on web-based self-help tools, frequently asked question listings, and subscription-based email lists, including a general R help email list, an R developer list, and an R package developer list. Although these models provide various support channels, Linux and Cytoscape mainly rely on dedicated channels (Linux: LF JIRA [66]; Cytoscape: a specific help desk [67]).

Not all support models for OSS are free. For example, ITK has a 3-way support: (1) ITK’s discourse forum enables discussion and mutual help among users, and dedicated volunteers usually provide detailed example codes [68]; (2) the NIH has continued to provide maintenance contracts for bug fixes, incremental improvements, and a moderate level of user support (maintenance has typically been performed by Kitware (Kitware, Inc), providing continuity and expertise); and (3) Kitware also offers commercial ITK support for a fee. Another example is that of Globus, which provides free support lists, operates a ticketing system [69], and guarantees subscribers a 1-business day response time on support tickets.

Surprisingly, free support is often available in a timely manner. One good example is the 3D Slicer, which had >13,000 forum posts in 2018, with an average response time of <2 days (or <8 hours during weekdays). For 3D Slicer, support may be provided either by the core developers or by experienced members of the user community. Public forums can be extremely active; for example, Bioconductor has >100 visitors per hour.

### Ecosystem Collaboration

Ecosystem collaborations are usually organized by working groups, conferences, networks, and community forums. Limited public information is available on how well OSS projects collaborate with other projects.

### Security

Security is important for biomedical software tools, as they are often used to manage and process patient data. To protect patient privacy, i2b2 provides secure remote access to patients in institutions through web services that anonymously list the number of patients in each institution [21]. Globus has maintained a strong security model for many years, using standards-based components and protocols that address message protection, authentication, delegation, and authorization for distributed infrastructures. Globus’s authorization is based on well-established standards, such as OAuth 2 and OpenID Connect, and leverages a federated log-in system to allow user authentication using one of the many supported identity providers (eg, institutional identities, eRA Commons, ORCID, and Google [Google, Inc]). The Globus high assurance tier provides additional security controls to meet the higher authentication and authorization standards required for access to restricted data, such as protected health information. Data transfers can be encrypted using OpenSSL libraries, and communication channels with the Globus service are Transport Layer Security 1.2 encrypted.

Linux has strong security features and is widely used outside biomedical domains. The Linux kernel allows administrators to improve security at the lowest level by modifying the attributes of the kernel’s operation, building additional security measures into the kernel to avoid common buffer overflow attacks, and setting different access restrictions for different kinds of users [70]. In addition, there are many Linux security extension enhancements, such as ExecShield and Position Independent Executable [71]. The other examined OSS use cases did not provide detailed information publicly about security. However, security enhancement should become the focus of future releases of research software.

### Legal Concerns

Among the 10 OSS use cases, a popular licensing model is the GPL, which allows the distribution and sale of modified and unmodified versions but requires that all the copies be released under the same license and be accompanied by the complete corresponding source code. For example, Linux was released under GPL version 2, whereas R and tranSMART used GPL version 3.

It is also feasible to use different licensing models for different components of an OSS. For example, Bioconductor packages belong to multiple license groups: artistic license version 2, GPL, Massachusetts Institute of Technology (MIT), Berkeley Software Distribution (BSD), and creative commons licenses that have minimal requirements regarding how the software can be redistributed [72]. Globus also uses mixed licensing models. The client-side software is licensed under the Globus community license, which allows subscribers to access the source code for the purposes of code review and contribution, whereas the software operated by Globus as a service is not licensed.

Open-source licensing models used by the other OSS use cases include Apache 2 (OHDSI and ITK), Mozilla Public License version 2.0, with the Health care Disclaimer addendum (i2b2) [73], and GPL version 3 (tranSMART). REDCap requires a nonprofit end user license agreement between an institution and the Vanderbilt University, and its code base is not open to an individual developer. Finally, the 3D Slicer license, although generally highly permissive, is not a standard Open Source Initiative certified license. Instead, it is a custom license that was defined via coordination with the legal department of Brigham and Women’s Hospital, which primarily aims to mitigate liability risks because of the nature of the application (visualization and analysis in support of research applications on clinical images).

### Financing

Of the 10 OSS use cases, 8 (80%) started with federal research funding. For example, Bioconductor began receiving the NIH National Human Genome Research Institute’s support in 2003 and NCI/ITCR funding in 2014. The 3D Slicer has received direct or indirect support from many research grants (primarily NIH) over the course of several decades [74] but no sustained funding from any single source or program. Cytoscape received support from the National Institute of General Medical Sciences and the National Resource for Network Biology. REDCap received early support from the National Center for Research Resources. The early development of Globus was supported by the National Science Foundation and the Department of Energy, whereas more recent work on high assurance mechanisms has been supported by the NIH. Federal research funding is vital, as it encourages research on OSS to focus on scientific explorations and research ecosystem development. At the same time, although grants guarantee the researchers money to experiment, researchers still have to look for sustainable solutions beyond the grant cycle [29].

Industry and membership support are common in mature OSS cases. For example, premium Globus features (eg, data sharing, use reporting, and guaranteed support levels) are offered to institutions under an annual subscription, which is a flat annual fee based on the institutions’ level of research activity. Linux continues to be supported by individual memberships (thousands of members) and annual corporate memberships (>1000 corporate members) [75]. The R Foundation is largely supported by members (membership fees from supporting persons, institutions, and benefactors) and *one-off donations*.

Multiple sponsor programs involving both academic and industry sponsors are also feasible. For example, ITK has continual funding from the NIH for maintenance to enable its free use and, at the same time, has commercial-grade support. OHDSI also has both private and public funding support. The i2b2 tranSMART Foundation has 4 sponsorship programs: contributing sponsors, corporate sponsors, sustaining sponsors, and event sponsors [76]. Through the tranSMART and the successor i2b2 tranSMART Foundation efforts, Keith Elliston and colleagues started Axiomedix (Axiomedix, Inc) in 2018 specifically to provide a commercial (for-profit) support mechanism for government-funded OSS. Axiomedix offers a 4-part business model that helps to support and sustain the open-source platforms: first, a commercial-grade software publishing and support model; second, a full-service solution offering for these supported platforms that includes installation, configuration, data loading, curation, and more; third, a software development and customization model (the Axiomedix Expert Network) that enables core open-source developers to take up contracts and consulting for customers; and finally, a model for developing new products and platforms that leverages open-source tools, a network of experienced open-source developers, and the knowledge of subject-matter experts to develop new open-source or commercial tools.

### Marketing

The 10 OSS use cases have a variety of marketing channels, including the use of logos (3D Slicer, Globus, and i2b2 tranSMART), websites (3D Slicer, Bioconductor, Globus, and i2b2 tranSMART), mailing lists (Cytoscape, Globus, and i2b2 tranSMART), forums (3D Slicer, Cytoscape, and i2b2 tranSMART), Twitter (Twitter, Inc; 3D Slicer, Bioconductor, Cytoscape, Globus, and i2b2 tranSMART), LinkedIn (LinkedIn, Inc; Globus and i2b2 tranSMART), Facebook (Meta Platforms, Inc; i2b2 tranSMART), YouTube (Google, Inc; 3D Slicer, Bioconductor, and i2b2 tranSMART), Tumblr (Tumblr, Inc; Cytoscape), Vimeo (Vimeo, Inc; Cytoscape), and Pinterest accounts (Pinterest, Inc; Cytoscape).

Additional channels include conferences, workshops, and publications. For example, the ITK is introduced at medical imaging conferences. R gains market share through an *evangelist* approach among statisticians, data analysts, and others from the biomedical community. Moreover, surveys administered to collect user feedback also act as a form of marketing. For example, the 3D Slicer team conducts small-scale surveys on forums and collects feedback forms during training courses. Similarly, the Globus team conducts surveys during workshops and tutorials.

### Dependency Hygiene

Of the 10 OSS (all except R), 9 (90%) have many dependencies on other packages. Bioconductor and OHDSI depend on many R packages, and REDCap depends on MySQL, whereas Cytoscape relies on external services, including cxMate. As dependencies may complicate installation and use, i2b2 provides Docker containers for easy installation [77]. Software models mainly provide dependency information through documentation, for example, installation guides; however, few models describe the license and security status of each dependency. Crichton [78] points out the potential danger of complicated dependencies, warning that “Blackbox can make it difficult to see that there are far fewer maintainers working behind the scenes at each of these open-source projects than what one might expect.” Thus, it is critical to provide transparent information about the dependency tree of the code libraries. The 3D Slicer is a good example, as it provides an extensive list of dependencies that is publicly available.

## Discussion

We discussed 10 representative OSS use cases that have demonstrated sustainable practices, particularly in the biomedical domain. Although not a comprehensive list, these examples highlight the following as essential attributes of successful OSS development: alignment with unmet scientific needs, a dedicated development team, a vibrant user community, a feasible licensing model, a sustainable financial model, and effective product management.

### Alignment With Unmet Scientific Needs

At the inception of an OSS project, it must identify and meet important scientific needs instead of complying with mandatory rules or obtaining external financial rewards [79]. Meeting these needs gives the software its *soul*, that is, its unique identity. For example, Cytoscape fulfills the need for a visualization tool to represent complex interactions among molecules, Bioconductor reduces the barrier to entry involved in the effective use and sharing of computational biology and bioinformatics tools [57], and Globus addresses the need for frictionless data transfer and sharing. As the scientific community’s needs are diverse and dynamic, developers should consider the potential expansions beyond the first application and adopt a highly reusable infrastructure even at the initiation stage.

### Dedicated Development Team

An OSS project should have a core development team, which has not only developed an initial version of the software but will also continue to be committed to future versions. The team is the *brain* of the software and its intellectual center. For example, Globus includes services for identity management, data transfer, data sharing, and group management; interfaces such as application programming interfaces, web apps, and a command-line client; and software to manage data access on >10 distinct storage platforms and file systems. Only a dedicated and highly experienced development team can put all these components together in a concerted fashion.

However, maintaining such teams can be difficult. According to Atiq et al [12], the motivations of developers usually include both intrinsic (eg, creativity and fun) and extrinsic aspects (eg, financial rewards, development of job-related skills, and peer recognition). Atiq et al [12] further pointed out that transparent and fair extrinsic rewards and effective and open communications among developers are key characteristics for ensuring the long-term sustainability of OSS projects.

More importantly, the whole research community needs to realize that the creation of a dedicated development team is incredibly difficult if that team cannot gain recognition for their contribution. Unfortunately, it is still true that, in academia, the effort invested in the development of software is often not recognized as important and is certainly viewed less favorably than traditional research activities.

### Vibrant User Community

To be successful, an OSS project should also have a vibrant user community whose organizational structure and ongoing activities can facilitate communication both among and across the developer and user groups. This community would foster the *materialization* of the value of the software while specifying the functionality requirements for future versions. A vibrant user community represents the *heart* of the software, which drives the development cycle. For example, 3D Slicer and ITK have large and stable user bases, mainly in the radiology and biomedical imaging communities. OHDSI tools have large user bases in the clinical informatics and population health informatics communities. Moreover, we highly recommend engaging scientists outside the original team and involving a broad array of stakeholders. In addition, we support encouraging the *diaspora effect*, where postdoctorates and students who move on to other institutions continue using the software used or created by their original group.

It is also important to realize that the *users* of enterprise-level OSS are institutions, not individual researchers. In fact, Masys et al [79] defined successful adoption as at least 50% of the intended institutions adopting and implementing a tool. They suggested that, instead of a one-size-fits-all technical approach, developers should provide flexible local implementations and customizations, such as the optional use of terminology standards. This flexibility is essential for building a vibrant user community and facilitating successful adoption.

### Feasible Licensing Model

A sustainable OSS project also needs a licensing model that fits the nature of the software, its distribution channel, and stakeholder interests. A licensing model resembles a *skeletal system*, providing a framework for the software to function legally.

OSS licensing generally falls into 4 categories: nonpermissive, weakly permissive, fully permissive, and noncompliant. Open-source licenses are evaluated as to whether they conform to the Open-Source Definition by the Open Source Initiative, a 501c3 nonprofit established to be a steward of open-source licenses.

Nonpermissive licenses, such as the GPL and the Affero GPL, not only allow commercial and noncommercial reuse but also require the release of all modified code and any external code linked to this code. The most well-known example is Linux, which is now under GPL version 2. Without the use of lawyers, its founder, Linus Torvalds, wrote a brief license stating that no fee may be charged for its distribution. As internet-based delivery systems were in an early stage of development, this move eliminated the *floppy drive mills* whereby individuals or companies could send copies of Linux to consumers for a fee. As the goal was not to allow others to make money on *free software distribution* at the time of writing, the model fit. When OSS code was to be modified or added on to, several open-source licenses were created and evolved. GPL version 3 (used by R and tranSMART) is the most restrictive open-source license, which requires that any enhancements (such as new features) incorporated into the software must be released along with the source code. Commercial software companies refer to the GPL version 3 as a *toxic license*. Once the software contains any GPL version 3 codes, its future licensing and that of all other software that carries it would be forever under the GPL version 3 license. The *infectiousness* keeps commercial companies away from using GPL code in their products; however, it could be one of the most important reasons why R is widely used and is successfully evolving. From our point of view, nonpermissive licenses may fit best for software that is fundamental to essential scientific discovery and highly used by researchers from very broad domains and where funding support may mostly come from noncommercial sources.

Weakly permissive licenses (eg, Mozilla Public License 2) allow commercial and noncommercial use and require release on a file-by-file basis for any modified code. Fully permissive licenses provide unrestricted reuse of code for commercial and noncommercial purposes. Fully permissive licenses include the Apache 2, MIT, and BSD licenses, among others. One of the main motivations of the popular Apache 2 license was to enable the ability to integrate open-source code into a project without having to release any enhancements to the code, that is, the ability to *build on the shoulders of giants*. Finally, many projects that are considered open-source release codes under custom licenses are non–Open Source Initiative compliant. Thus, although these projects may make the code available, they cannot be considered open-source compliant.

There is a slow migration in the research software field toward fully permissive licenses because of limited commercial support. Elster [80] discusses how the license of research software may have an impact on obtaining industrial funding support. Many informatics technology companies choose research software with full permissive licenses over nonpermissive licenses, as nonpermissive licenses add restrictions to code reuse in commercial software, raising concerns about future commercialization. BSD license, as an example of a fully permissive license, allows the inclusion of open-source code in commercial code. On the other hand, some companies prefer nonpermissive licenses to fully permissive licenses, as they do not want their competitors to build commercial code on top of the OSS that those companies previously funded. Although this type of self-interested licensing prevailed in the early days of the software industry, the industry soon realized that having tens or hundreds of groups reinventing the same code was limiting the progress of the industry. As a result, there has been a wide and growing adoption of fully permissive licenses such as MIT and Apache [81].

Software licensing creates a binding agreement on the way a licensee may use or distribute the programs or codes. Just as a software-wrapped or click-through user licensing agreement is binding, so too is the use of OSS and code. When a research software is commercialized, a *free version* for academic use may be kept; however, if it is used outside the terms of that license, a *commercial license* must be purchased. Thus, the environment of the use of the software can play an important role in whether a user is in violation of the applicable license. A violation may result in harsh additional fees or even legal actions.

### Sustainable Financial Model

An OSS project requires a sustainable financial model (formal or informal) that can keep the software and its user community moving forward. A sustainable financial model is a part of the *circulatory system*, supplying *blood* to sustain the software ecosystem. The i2b2 tranSMART, Globus, and Linux are excellent examples that leverage multiple types of sources to sustain software development.

The public–private partnership is becoming a feasible way to support an OSS project in the long term; however, the establishment of these partnerships may not be easy. Industry partners usually have concerns regarding profitable commercialization time. The public release of an OSS project, including its knowledge and source code, may allow the market competitors to catch up quickly, as opposed to traditional commercialized software business practices, where intellectual property is commonly concealed as long as possible. However, at the same time, an OSS project may quickly attract a large number of outside users and new developers whose contributions can improve the robustness of a product, enabling platform-based customizations across multiple institutions. Robust implementations and large user bases increase the commercial potential of OSS projects.

Along with the development of OSS, its financial model can change over time. Globus has tried a mix of several financial sustainability strategies: relying on grant-based federal funding, offering free OSS, forming an international research consortium, launching a commercial company, and forming an industry organization [82]. Globus found that many activities critical to sustaining software are outside the mission of federal funding agencies. Few developers have the freedom to contribute to a software project that addresses the research community’s needs at large and does not directly advance the contributor’s own mission. Contributors are not always able to provide ongoing maintenance or user support for the code they contributed, much less for the rest of the code base. As copies (*forks*) are maintained by separate teams, new features may no longer be shared with the entire community, and user requirements between the nonprofit research community and the industry do not always align. After 15 years, Globus pivoted to a sustainable model of providing free, cloud-based software-as-a-service to researchers and premium subscriptions for institutions. Focusing the primary software product on the needs of researchers and the revenue mechanism of creating value for resource providers is proving to be a viable financial model for sustaining Globus.

In the literature review section, we summarized a gamut of financial models for long-term software sustainability. Each approach has its own strengths and weaknesses. For example, community-based sustainability (eg, the not-for-profit organization model mentioned in the literature review), including appropriate forking of branch-development efforts, is in many ways ideal as it leverages the collective and continuous efforts of entire communities. However, it might not be appropriate for important niche areas of development; it might overemphasize broad adoption rather than quality, novelty, or significance, and it might not be able to leverage efforts that do not follow the same open-source licensing structure. Commercialization (eg, content licensing model, user subscription model, freemium model, razor and blades model), such as the adoption of software modules in clinical workstations, leverages a large pool of resources and software libraries in addition to creating a direct path to a broad user base willing to pay for it. However, commercialization is limited by proprietary restrictions and by its dependency on profit-making motives, which might not align well with biomedical significance or with *investment for the future* policies. Various infrastructure-based models (eg, macro research and development infrastructure model) can be effective ways to pool resources and avoid replication; however, they depend on a decision mechanism for the selection of the small percentage of software products that would be supported. Moreover, infrastructure-based models might be less prone to supporting innovation because of their not-so-dynamic nature. Various funding-based mechanisms (eg, national funding model and institutional support model) combine the advantages of dynamic selection and evolution of software products through the process of merit-based reviews. Unfortunately, they are limited by the harsh reality that existing funding is far less than the cost of long-term maintenance of meritorious software, a situation that is unlikely to change in the foreseeable future.

### Effective Product Management

Finally, an OSS project requires effective product management, which is a part of the *neural system*, enabling fast communications between the *brain* and other systems.

Roadmaps outline the development status of projects, including both the dates of past events and future events, so individuals can understand the speed, goals, and activities of specific projects, thereby improving sustainability through well-conceived deadlines and structures [83]. We found that 6 of the OSS cases on our list had well-designed roadmaps. The design of a road map is usually an evolving process that requires multiple rounds of internal discussions as well as extensive communication with the community of users and external developers. R and Linux offer a road map publicly. It is possible that these 2 OSS tools rely highly on the developer community’s contributions, whereas the community makes its own decision about what it thinks is important, showing its partial resistance to central planning.

Although a road map designates the plans, it is the software release that shows the actual achievement. As OSS often involve the participation of a large number of external developers, the coordination of software releases can be more complicated. R provides a very good example by providing extensive support to facilitate external developers’ package testing and release. Regarding the release strategy, OSS communities adopt either feature-based or time-based releases [84]. A feature-based release strategy is more often adopted by early-stage OSS projects. As an OSS project grows in size and complexity, it may move to time-based release, which helps prioritize development activities.

With regard to OSS quality assurance, a large user community may provide the project with good coverage in terms of bug hunting, performance, and scalability testing; however, most users do not consciously explore uncharted edge functionalities and thus leave certain bugs unfound [84]. Therefore, it is recommended to have professional testing and share a core bug report with the public through a public ticket tracker [85]. Moreover, an OSS project needs a version control system to coordinate release management, bug management, code stability and experimental development efforts, interdeveloper communication, and the authorization of changes by particular developers [86]. Public information shows that most of the 10 OSS use cases discussed go through rigorous testing.

As the *instruction manual for software* [87], documentation is essential in creating a sustainable community, as it allows users and external developers to rapidly become familiar with the software and use it for their own projects. Therefore, documentation is a key way of creating smoother internal transitions among generations of core developers. When familiarizing themselves with the OSS through the documentation is not enough for new users and external developers, specific support is essential to engage them, such as answering questions in a public forum. As mentioned in the *Results* section, all 10 OSS use cases provide comprehensive documentation and various types of support to users and new developers.

### Strengths and Limitations

The selection of the examined software products was completed by 13 participants, comprising a group of people with rich knowledge of the sustainability of OSS tools and the promotion of industry partnerships. Although we conducted a comprehensive analysis of 10 aspects of the selected OSS use cases, there appears to be a risk of biasing the paper’s findings toward the interests of the ITCR working group and overlooking potentially important sustainability models. Limited to publicly available information, we were not able to discuss failed OSS examples and important checkpoints. Our future goal is to conduct a survey of a much broader research community to continue these discussions.

In addition to the information discussed about general OSS aspects using the Nesbitt list [28], we would like to briefly discuss other important aspects of research software, such as scientific accuracy and reproducibility, compliance, and ethics and integrity. Rougier et al [88] defined reproducible software as the publishing of software and data as a product of the used software, its related data, and the articles involved. For software to be reproducible, its source code must be investigated, and its models must be documented thoroughly and precisely. Buck [89] explains that to improve reproducibility, transparency must be a top priority, despite the interference of high cost. To increase transparency, free OSS provides other scientists (besides software developers) with cheap options to validate their reported results and further apply this open science framework to other scientific research activities. Another aspect, compliance, is also critical for OSS, as the software may be incorporated into commercial uses, used to raise awareness about compliance, or used to display specific cases of noncompliance [90]. When distributed to external sources, the OSS licenses must be reviewed before compliance can be achieved (eg, for sharing, license fees, and compatibility purposes). Finally, ethics and integrity are essential for software in biomedical research. The use of OSS should allow researchers to meet the professional standards of practice, and the use of OSS must align with the 4 basic principles in the field: nonmaleficence, beneficence, autonomy, and justice.

### Other Initiatives and Future Perspectives

In addition to the NCI ITCR, several informatics efforts across the NIH have also emphasized creating an approach to OSS sustainability. The National Center for Advancing Translational Sciences, Clinical and Translational Science Awards links with programs from the NIH Office of the Director, including the Big Data to Knowledge (BD2K) [91,92] and the Data Science program [93], and most recently, the All of US Precision Medicine Initiative [94]. BD2K is a trans-NIH initiative launched in 2013 to support the research and development of innovative and transformative approaches and tools that maximize and accelerate the integration of big data and data science into biomedical research. BD2K recognizes that software is a necessary part of any modern solution to biological problems. Representing the shared interest of the national Clinical and Translational Science Awards consortium, the National Center for Data to Health is particularly interested in sustainability strategies for data management infrastructure, which again inevitably involves the sustainability of software tools revolving around clinical data.

Other countries, such as the United Kingdom and Germany, are also making national policies to improve software sustainability. Currently, the United Kingdom has developed a research and innovation road map and is using the *research and development system* as a connection to sources of funding that can flow to universities, research institutes, government laboratories, charities, and businesses [95]. The United Kingdom is moving toward minimizing bureaucracy in the public funding system to keep checks and approvals that will effectively manage public money and make informed decisions for the system. Moreover, the United Kingdom is increasing clarity and coherence in research and development funding to allow researchers to have confidence in long-term investments and enable agile funding to allow the system to tackle issues of national priority and urgency. For biomedicine, scholars in the United Kingdom recommend OSS in health care information systems to improve safety and effectiveness [96]. Similarly, Germany has created a more unified software policy [97] and has outlined the following recommendations: (1) in its foundation, research software must have an open-source code, as well as trustworthy, supportive, and appropriate infrastructure and infrastructure facilities; (2) senior researchers and research managers must develop good scientific practices, and there must be a general shift toward the acquisition of central licenses rather than commercial software and services; and (3) in the provision of research software, there must be a shift from the role of developer to the role of provider. There are still many challenges at the organizational and technical levels related to the development, use, and provision of research.

Looking forward, it will be important to learn from international governance examples and engage with other groups interested in sustainable software models. One notable community is the Workshop on Sustainable Software for Science: Practice and Experiences (WSSSPE) [98], a workshop series aimed at promoting sustainable research software by focusing on principles and best practices, careers, learning, and accreditation. The fourth WSSSPE created a group interested in writing white papers that focus on scientific environments and their implications, targeting developers and project managers of research software. Another notable community is the Science Gateways Community Institute, which provides consulting services for sustainability and business planning [99].

### Conclusions

#### Overview

Our review of the existing sustainability models and 10 OSS use cases strongly confirms the importance of the 3 proposed future focus areas of the SIP-WG: (1) to develop a workflow or decision tree to support informed decision-making that is consistent with ITCR expectations and the future licensing needs of open-source tools; (2) to provide a consultancy service for the 10 sustainability aspects, especially governance, licensing, code quality, and community building, in collaboration with the ITCR program; and (3) to develop a collection of business model archetypes that can be used as starting templates to formally document the dissemination and sustainability plans for new software development initiatives. In addition, we stress on 5 important actions that should be considered in future ITCR activities, as described in the following sections.

#### Discussion of the Feasibility of Sustainability Models for ITCR Projects

An important agenda item of the SIP-WG's future work should be a discussion of the feasibility of various sustainability models for the many ITCR support projects, including nonprofit models (eg, the not-for-profit organization model, the national funding model, the infrastructure model, the institutional support model, and the donation model), and commercial models (eg, the distributed network model, the content licensing or industrial support model, the user subscription model, the freemium model, the split licensing model, the razor and blades model, the macro research and development infrastructure model, and the mixed model).

#### Exploration of the Potential Licensing Models

The licensing of research software will have a direct impact on public–private partnerships. A mixed licensing model may be the best way to strike a balance between free use (for broad use) and paid use (for funding support). Given the potential complexities of different OSS approaches, key stakeholders should consider the licensing structure of their software models as early as possible. Important decisions and changes must align well with the road map of software development and maintenance, as changing the licensing of existing projects can be very challenging. Once an open-source project integrates code from external contributors, it becomes logistically difficult to legally change the licensing on the code.

#### Provision of Reward Mechanisms to Enhance Stakeholders’ Motivation to Focus on Sustainability

The WSSSPE community has pointed out the importance of enhancing stakeholders’ motivation through credits and rewards [98]. Currently, the main credit given for developing a research OSS is through publications. Key contributors should be encouraged to list the creation of software resources on their resumes and further value the OSS in the grant funding review process. We should also provide reward mechanisms to fairly allocate credit to external developers who have contributed to successful expansions and adoptions. Finally, universities and research institutions should create viable career paths for researchers developing software in academia to encourage them to continuously work on research OSS development.

#### Establishment of a Central Library to Make OSS Visible and Reusable

In addition, we should consider establishing a central library to make ITCR-funded OSS more visible and reusable for a large number of biomedical researchers. The open-access library should index the OSS tools with brief descriptions of their functions and simple examples. This library should point to the latest version of each OSS tool. It would especially serve as a repository for retired OSS tools, which may have short-term difficulties in obtaining funding support. Ideally, this library should be searchable, enabling something like a Google search for research OSS. When researchers have certain needs, they can first search within this library to find out if there is an existing tool available to meet their needs or if there is an existing tool they may expand upon to meet their needs.

Before establishing such a software library, we need to fully understand who the expected users of the library would be, what their incentive to use it would be, how often entries would be added and updated, whose responsibility would be to update the records, and what funding sources would support the future releases of a piece of software. Without continuous curation, there is an eventual risk that software libraries may become software graveyards.

#### Provision of Industry Standard Support

Finally, we should allocate consulting resources to research OSS projects (especially at the early stage of development), which can guide these projects to follow state-of-the-art industry standards on code quality control, ecosystem collaboration, security, and dependency hygiene.

## Appendix

Multimedia Appendix 1 Full descriptions of open-source software use cases.

## Abbreviations

BD2K: Big Data to Knowledge
BSD: Berkeley Software Distribution
GPL: general public license
i2b2: Informatics for Integrating Biology and the Bedside
ITCR: Informatics Technology for Cancer Research
ITK: Insight Toolkit
MIT: Massachusetts Institute of Technology
NCI: National Cancer Institute
NIH: National Institutes of Health
OHDSI: Observational Health Data Sciences and Informatics
OSS: open-source software
REDCap: Research Electronic Data Capture
SIP-WG: sustainability and industry partnership working group
WSSSPE: Workshop on Sustainable Software for Science: Practice and Experiences
