# Supplementary material for: Open-source Software Sustainability Models: Initial White Paper From the Informatics Technology for Cancer Research Sustainability and Industry Partnership Working Group
Source: J Med Internet Res. 2021 Dec 2;23(12):e20028. doi: 10.2196/20028 (PMC8686402; doi:10.2196/20028)
Supplement: Multimedia Appendix 1 [file jmir_v23i12e20028_app1.docx]

**Multimedia Appendix 1. Full Descriptions of Ten OSS Use Cases**

| **Table 2. Summary Comparison of Governance of Academic OSS** | |
| --- | --- |
| **Software tool** | **Characteristics** |
| **3D Slicer** | - Decision making is typically happening on the GitHub pull requests, user/developer hangouts and in-person meetings; decisions are publicly documented via GitHub, forum discussions, and Wiki pages; Roadmap for the development is publicly available. - Contribution guidelines are publicly available; core developers and users meet in person twice a year; sub-communities have regular meetings. - Feasibility studies and experimental designs are documented on “Labs” pages. - Forum and video conference meetings are publicly available; source code is in GitHub; bug tracker is publicly available; training courses are organized at major conferences and educational institutions. |
| **Bioconductor** | - Technical advisory board meets monthly to develop strategies to ensure long-term technical suitability of core infrastructure and to identify funding strategies for long-term viability. - Scientific advisory board includes external experts, providing annual guidance. |
| **Cytoscape** | - Cytoscape is architected as core software augmented by Cytoscape Apps. - Core team contributes to the core software through GitHub commit. - Cytoscape 3.0 has a clearly defined, simplified API. - Each class in the public API maintains an explicit backwards compatibility contract so that both core developers and app writers understand how this class might change. |
| **Globus** | - Technical and business decisions are in the hands of the Globus team. - Institutional subscribers via subscriber meetings and consultations, and community users via mail lists, webinars and on-site tutorials, provide input on feature prioritization, product roadmap, subscription pricing, and sustainability models. |
| **i2b2 tranSMART** | - The i2b2 tranSMART Foundation was established; the bylaws are available publicly. - The foundation includes high-level leaders, membership program, and working groups, communicating through mail list and recorded online webinars. |
| **ITK** | - ITK consortium manages licensing, owns the copyright, and acts as a governing body. - Code changes are strictly controlled by limiting the commit of repository changes. - A code review process has been established for submissions. - As a forum for proposed changes, an online ITK journal documents changes and acts. - Development is maintained fully via GitHub. - Contribution guidelines are documented. |
| **Linux** | - Linux is the premier example of open source sustainability and success. - Linux Foundation was founded in 2000 as a neutral home, providing fellowships, IT operations, training, and events. - Board of directors is comprised of 22 senior leaders from across the IT industry. - Board members represent Linux Foundation members and the Linux developer community. |
| **OHDSI** | - OHDSI has an international network, centrally coordinated at Columbia University. - OHDSI hosts a collection of open source tools, among which OMOP related standard vocabulary are foundational tools. |
| **R** | - Since mid-1997, there has been a core team responsible for overseeing its development. - R Foundation, a registered association under Austrian law, is currently hosted by the Vienna University of Economics and Business; R Foundation supports the development, teaching and training, and the organization of meetings and conferences. - Ordinary members of the Foundation are elected by a majority vote of the general assembly. - New ordinary members are selected based on their non-monetary contributions. |
| **REDCap** | - Consortium consists of non-profit organizations interested in expanding functionality. - Each partner institutional is hosting as a “platform” to reach out and bring value to its intended end users; site was given access to the codebase; code is available at no charge to institutional partners and is restricted in use, permitted only for non-commercial research purposes. - Vanderbilt is the only entity that can distribute it; all derived works – such as innovations or programmatic features added on by the user – are owned by Vanderbilt. |

| **Table 3. Summary Comparison of Documentation of Academic OSS** | |
| --- | --- |
| **Software tool** | **Characteristics** |
| **3D Slicer** | - Documentations include Wiki, ReadTheDocs, crowd-sourced documentation, various recipes, and YouTube videos. - Tutorials page with slides and sample datasets materials are publicly available. - Commit style guidelines are documented publicly, with human-focused readable notes. - It does not have support for internationalization of the documentation, but there are user communities that developed documentation in their languages independently, e.g., in Chinese. |
| **Bioconductor** | - Three-level documentation is available: workflows, package vignettes, and function manual pages. - Users often become developers, making their own algorithms and approaches available to others. |
| **Cytoscape** | - Extensive user and developer documentation: developer resources include an issue tracker, Nexus repository, nightly builds, and code metrics. - Cytoscape “App Ladder” teaches essential skills for developing Cytoscape Apps. - Publicly available resources include basic and advanced tutorials, a YouTube channel, a blog of published figures, as well as several presentations. |
| **Globus** | - Fully developed documentation is available, including installation guides, API usage guides, FAQs, tutorials, and how to guides. - Each release is clearly versioned with release notes and change history. |
| **i2b2 tranSMART** | - i2b2 updates documentation through a Wiki page; developers commit messages in GitHub; i2b2 provides human-focused notes of changes of each release and bug fixes. - tranSMART provides documentation on version 16.3. |
| **ITK** | - ITK provide a user guidebook. - Coding examples are automatically built as part of nightly tests. |
| **Linux** | - Code is available through GitHub. |
| **OHDSI** | - Available documentation introduces how to get started with OHDSI, common Data Model, and ETL creation best practices, as well as tool specific information. |
| **R** | - R provides various fully developed documentation, adequately covering two types of development: writing R extensions and developing R itself (by providing internal structure and coding standards). |
| **REDCap** | - Detailed documentation is available for setting up and software usage, but not on contributing source code. |

| **Table 4. Summary Comparison of Code Quality of Academic OSS** | |
| --- | --- |
| **Software tool** | **Characteristics** |
| **3D Slicer** | - Code styling guidelines are documented publicly; new functionality is contributed by modifying the core application or by submitting an extension (code styling guidelines are not enforced for extensions). - Developers conduct about 700 tests for the core application and testing results are available publicly; extension contributors manage the test for extensions (not verified by core developers). - Contribution process is documented publicly. |
| **Bioconductor** | - Packages are built nightly on Windows, macOS, and Linux platforms. - Packages are built successfully before propagating to public user-facing repositories. - Tests are conducted to ensure overall package integrity and integration with current versions of package dependencies. |
| **Cytoscape** | - Core developers use Jenkins and open source automation tool to build and test software projects continuously. - Contributors on external extensions are strongly encouraged to thoroughly test apps before releasing them; the Cytoscape team does not independently review these apps. |
| **Globus** | - Professionally maintained and managed code base employs best practices, such as revision control in GitHub, extensive documentation, code comments, linting, distribution of knowledge across team members, and incremental/iterative software development. - Globus employs a continuous integration environment, automated tests, and documented, standardized human QA testing to ensure code quality. - Code is reviewed by at least one engineer other than the code author before being released to production. - Globus developers follow secure development practices, including OWASP recommendations to prevent web application security risks. |
| **i2b2 tranSMART** | - Both i2b2 and tranSMART have extensive automated and manual testing as a part of their well define release process. |
| **ITK** | - Automated nightly builds and tests are as far back as 1999. - Strictly enforced coding conventions with consistent naming rules. |
| **Linux** | - The code quality has not been discussed in case profiling. |
| **OHDSI** | - The code quality varies, because a large collection of tools is developed around the OMOP CDM. |
| **R** | - Guidelines are maintained for its Software Development Life Cycle; coding standards are established. - Apache Subversion is used to maintain current and historical versions of files; software development and testing methodologies are employed by R Core in order to maximize the accuracy, reliability, and consistency; some aspects are handled collaboratively, others are handled by members of the team with specific interests and expertise in focused areas. - R can run on a wide variety of UNIX platforms and similar systems (including FreeBSD and Linux), Windows and MacOS. |
| **REDCap** | - Source code does not open to the community. |

| **Table 5. Summary Comparison of Support of Academic OSS** | |
| --- | --- |
| **Software tool** | **Characteristics** |
| **3D Slicer** | - Support is provided by the community of developers and users; only a small fraction has resources for 3D Slicer development; even smaller fraction has dedicated funding. - For the over 13,000 forum posts in 2018, the average response time was less than 2 days (or less than 8 hours during weekdays). - Active members on several forums and mailing lists provide support via various social media platforms (e.g., Twitter, Research Gate, YouTube). |
| **Bioconductor** | - Individual “landing pages” (e.g., DESeq2) provides an overview of the package, installation instructions, and usage statistics. - User-oriented stack overflow-style support site is publicly available and active (100’s of visitors per hour; fast response). - All packages include maintainers’ email addresses and links to GitHub issues. - Developer supports are available by the email lists. - Hybrid community slack is available for experienced user / developer collaboration. |
| **Cytoscape** | - Cytoscape Help Desk |
| **Globus** | - Several support options are available, such as online self-help tools, listserv groups, and a ticket submission system with a responsive, dedicated support team. - Guarantees subscribers are guaranteed a response time of one business day to support tickets. |
| **i2b2 tranSMART** | i2b2:   - bug tracker - Google forum for installation help   tranSMART:   - tranSMART Wiki |
| **ITK** | - ITK has mailing lists; over its long history, ITK has had dedicated volunteers who would give detailed help to users even including example code. - ITK has discourse forum for discussions and mutual help among users. - NIH has continued to provide maintenance contracts for bug fixes, incremental improvements, and a moderate level of user support. - Maintenance has typically been performed by Kitware, providing continuity and expertise. Kitware also offers commercial ITK support. |
| **Linux** | - Linux support is provided through the LF JIRA. |
| **OHDSI** | - Community-based discourse forum - GitHub issue tickets |
| **R** | - Online self-help tools; - FAQ listings - Subscription-based email lists, including general R-help email list, the R-developer list, and R- package-developer list |
| **REDCap** | - REDCap is supported by Vanderbilt University. |

| **Table 6. Summary Comparison of Ecosystem Collaboration of Academic OSS** | |
| --- | --- |
| **Software tool** | **Characteristics** |
| **3D Slicer** | - 3D Slicer leverages the ecosystem of related open source projects. - More extensive list of dependencies is available publicly; developers interact on a regular basis with developers for most of the upstream dependencies and actively contribute bug fixes and feature improvements to the appropriate repositories/ - 3D Slicer has automated testing on all major platforms available to minimize the possibility of regressions and conflicts. |
| **Bioconductor** | - 1649 packages; over 1000 maintainers; over 29,000 mentions in literature; 500,000 unique IP addresses in 2018 |
| **Cytoscape** | - An active and diverse community of developers and users - Communication happens mostly via mailing lists and social media (Twitter, publications Tumblr). |
| **Globus** | - Large user base of over 20,000 users per year and 140 institutional subscribers. - Annual conference for users and subscribers. - Active community listserv groups for users, admins and developers. - Numerous webinars and on-site, two-day workshops occur every year at research institutions across the US. - Many grant-funded collaborations to apply, extend, develop, or innovate Globus services in the context of science drivers. - Professional services and customer engagement teams to enable collaborations and to facilitate application of Globus services to research data management needs. |
| **i2b2 tranSMART** | - Guidelines of submitting new contributions are publicly available. |
| **ITK** | - Over 200 developers contributing to the source code of ITK |
| **Linux** | - A strong and vibrant community: 13,594 developers from at least 1,340 companies have contributed to the Linux kernel since 2005; >1,600 developers contributed to each kernel version - A new major kernel release occurs every 9–10 weeks; kernel community merged changes at an average rate of 7.8 patches per hour over the past 15 months. - A thriving commercial ecosystem: 95%+ of the top 1M web domains, 80%+ of smartphones run Android, 98%+ of the top 500 fastest supercomputers in the world, most of the global markets, including NYSE, NASDAQ, London Exchange, Tokyo Stock Exchange. - Most consumer electronic devices run on Linux; 75%+ of cloud-enabled enterprises report is using Linux as their primary cloud platform; E-commerce giants Amazon, EBay, PayPal, Walmart, and more run on Linux. |
| **OHDSI** | - Consortium is organized by projects and workgroups; OHDSI has annual symposium and satellite events, and community forum. |
| **R** | - Two conference series: useR! (a user forum) and DSC (a developer platform) - R core team meets collectively and/or in smaller groups |
| **REDCap** | - Consortium has two tiers of users with distinct characteristics: institutional users and investigator users. - REDCap can be installed in a variety of environments for compliance with such standards as HIPAA, 21 CFR Part 11, and FISMA (low, moderate, high). |

| **Table 7. Summary Comparison of Security of Academic OSS** | |
| --- | --- |
| **Software tool** | **Characteristics** |
| **3D Slicer** | - Online resources are available via HTTPS; downloads are available via HTTPS. - No policy is used to enforce two-factor authentication for contributors. - Binary packages are built on highly secured workstations, managed by Kitware staff, and protected by strict multi-factor authentication. - 3D Slicer does not have specific consideration for addressing security aspects. |
| **Bioconductor** | - Security has not been discussed in case profiling. |
| **Cytoscape** | - A platform-independent open-source Java application is released under the terms of the LGPL. |
| **Globus** | - Globus has maintained a strong security model for many years, using standards-based components and protocols that address message protection, authentication, delegation and authorization for distributed infrastructures. - Identity and access management is based on OAuth 2, providing an advanced, user consent based delegated authorization model that allow applications and services to act on behalf of users and other services. - Encrypted data transfers use the SSL cipher configured on the source and destination storage systems. Communication channels with the Globus service are TLS 1.2 encrypted. - Globus developers follow secure development practices, including OWASP recommendations to prevent web application security risks. Globus meets higher security standards for access to restricted data, including HIPAA regulated data. - Security reviews by various external bodies, including code reviews by the NSF Trusted CI center and HIPAA risk and gap analyses by third party auditors. |
| **i2b2 tranSMART** | i2b2:   - Securable remote access is available through web services; random numbers are added to aggregate counts to protect privacy. - Security enhancement is becoming the focus of the next core release.   tranSMART:   - Protected study operation requires administrator approval. |
| **ITK** | - Security is not a primary consideration for ITK, which is typically embedded within broader systems. - ITK is not particularly vulnerable to cyber-threats. Being implemented in a low-level language (C++), some types of memory hacks may be possible. |
| **Linux** | - Linux has strong security features. - The Linux kernel allows administrators to improve security at the lowest level by modifying attributes of the kernel’s operation, building additional security measures into the kernel to avoid common buffer overflow attacks, and setting restrict permissions and access for different users. - In addition, there are many Linux security extension enhancements, such as ExecShield, and Position Independent Executable. |
| **OHDSI** | - Regular discussions are related to toolset security. - Apache SHIRO is used for securing the WebAPI component. |
| **R** | - An inherent security risk in malicious code is making their way unnoticed into the central repositories; there are a few published papers on the potential security risk of R and general guidelines on how to handle security issues. - No definitive information on security for privately reporting security issues - No provenance chain for releases compiled binaries - No reported vulnerabilities produced for security issues published within a reasonable timeframe |
| **REDCap** | - It has a secure web connection with authentication and data logging. |

| **Table 8. Summary Comparison of Legal of Academic OSS** | |
| --- | --- |
| **Software tool** | **Characteristics** |
| **3D Slicer** | - Non-restrictive (commercial use permitted) license - Not an OSI-approved license - Specific license is defined via coordination with the BWH legal department, primarily aiming to mitigate liability risks. |
| **Bioconductor** | - Almost all Bioconductor packages are licensed in a way that allows use by any entity without permission. - A small number of packages specify “academic only” use. - Bioconductor packages belong to multiple license groups: Artistic license v2 (commercially friendly); GPL (distribution of any modified code must make the source available); and MIT, BSD, Creative Commons (permissive licenses that have minimal requirements about how the software can be redistributed). |
| **Cytoscape** | - Consortium holds the intellectual property rights of Cytoscape (LGPL), Cytoscape.js (LGPL), NeXO (BSD or similar, in progress), and NDEx (BSD or similar, in progress). |
| **Globus** | - Globus uses mixed licensing models. - Select software components operated as a service and client-side software, such as the Globus Command Line Interface, the Globus Software Development Kit, and some versions of Globus Connect (the software that enables storage systems to be accessed via the Globus service) are made available under the Apache 2.0 license. - Some versions of Globus Connect are licensed under the Globus Community License, under which subscribers can access source code for the purposes of code review and contribution. - The remaining software components operated as a service by Globus are not licensed. Globus holds no patents on Globus technology. |
| **i2b2 tranSMART** | - The i2b2 software is licensed through the Mozilla Public License (MPL) version 2.0 under the terms of the Healthcare Disclaimer addendum. - The tranSMART software is available under the terms of the version 3 of the GNU General Public License. |
| **ITK** | - ITK is licensed under Apache that requires contributors to abide by the open source requirements. - ITK does not have no embedded third-party packages that have licensing restrictions. - Patented materials were no longer permitted for inclusion. |
| **Linux** | - Linux is released under GNU General Public License (GPL) v2, which allows distribution and sale of possibly modified and unmodified versions but requires that all those copies be released under the same license and be accompanied by the complete corresponding source code. |
| **OHDSI** | - OHDSI packages have open Source licenses, such as Apache License 2. |
| **R** | - R is under the terms of the Free Software Foundation’s GNU General Public License. |
| **REDCap** | - Non-profit End-User License Agreement is made between an institution and Vanderbilt University (“Vanderbilt”), a not-for-profit corporation duly organized and existing under the laws of Tennessee. |

| **Table 9. Summary Comparison of Finance of Academic OSS** | |
| --- | --- |
| **Software tool** | **Characteristics** |
| **3D Slicer** | - No dedicated organization - 3D slicer is supported by academic groups and commercial entities, either choosing voluntarily to contribute resources to 3D Slicer development or applying/allocating research or product development funding. - Numerous research grants (primarily NIH) - Enforcing disclosure of financial support would not be desirable, as commercial entities often do not want to publicly acknowledge what open-source software they use in their products, or development of what features they actively support. |
| **Bioconductor** | - Initial support was from institutional funding. - NIH/NHGRI support began in 2003 - NCI/ITCR funding began in 2014; - Additional grants included the NSF, European Union, NCI ITCR, NHGRI, and the Silicon Valley Foundation. - Details are available in annual reports - Funding sources are almost never acknowledged in contributed packages. |
| **Cytoscape** | - Funding for continued development and maintenance of Cytoscape is provided by the U.S. National Institute of General Medical Sciences. - User support, education and new initiatives are supported by the National Resource for Network Biology. |
| **Globus** | - Globus is provided to the community via a freemium model. - Researchers may use Globus services for free. - Institutions pay a flat, annual subscription fee, based on the institution’s level of research activity, for unlimited use of premium features. |
| **i2b2 tranSMART** | - The foundation has four sponsorship programs: contributing sponsors, corporate sponsors, sustaining sponsors, event sponsors - Through the tranSMART and the successor i2b2-tranSMART Foundation efforts, Keith Elliston and colleagues started Axiomedix in 2018 specifically to provide a commercial (for profit) support mechanism for government funded open source. |
| **ITK** | - ITK is free to users. - NIH has provided continual funding for maintenance. - Commercial-grade support can be purchased. |
| **Linux** | - Foundation is a membership organization based on different levels of membership: individual memberships (over thousands), corporate annual membership (over 1,000 corporate members) |
| **OHDSI** | - OHDSI is not specifically funded by any single organization. - Funding sources have included private and public sources. |
| **R** | - R is funded largely by supporting members and “one-off donations.” - Annual membership fees are gathered from supporting persons, institutions, and benefactors. |
| **REDCap** | - Early support was provided by NCRR. |

|  | |
| --- | --- |
| **Table 10. Summary Comparison of Marketing of Academic OSS** | |
| **Software tool** | **Characteristics** |
| **3D Slicer** | - Logo, web site, publications, YouTube channel, and Twitter - There is no “marketing strategy” or dedicated personnel to support project marketing. - Official announcements of releases and major events are regularly made via forum. - Small-scale surveys are conducted on forum to get general feedback. - Information is also collected via feedback forms and during training courses and thematic discussions during project weeks. |
| **Bioconductor** | - A consistent web presence and annual conferences - Social media (e.g., twitter) and video sharing (YouTube) channels |
| **Cytoscape** | - Marketed through Twitter, Tumblr, Vimeo, and Pinterest - Mailing lists and forums for specialized audiences. |
| **Globus** | - Memorable name, unique logo, public website optimized for search - Participation at industry events - Social media (Twitter and LinkedIn) - News media via occasional press releases and article postings - Blog with frequent announcements and articles; - Email announcements, public workshops and user conferences, annual user conference - Enthusiastic user evangelists - Partnered with several commercial and non-commercial organizations |
| **i2b2 tranSMART** | - Logo, trademark - Official announcements available through official website - Workgroup mail lists - Administrator and developer mail lists - Social media (Twitter, LinkedIn, Facebook, Google forum; YouTube) |
| **ITK** | - Promotion at medical imaging conferences |
| **Linux** | - As of March 2016, top corporate contributors to Linux kernel include Intel, Red Hat, IBM, Motorola, Linaro, Google, Mellanox, SUSE, AMD, Renesas Electronics, Samsung, Rockchip, Oracle, ARM, Canonical, Broadcom. - The Linux system is available as a product from Red Hat, which provides storage, operating system platforms, middleware, applications, management products, and support, training, and consulting services. |
| **OHDSI** | - Not a business entity - Not actively engage in marketing - Specific workgroup is focusing on dissemination. - Researchers provide acknowledgment to OHDSI in publications and presentations. |
| **R** | - No direct information on marketing strategies - R gained market share by an “evangelist” approach amongst statisticians, data analysts and others from the biomedical community. - Some major commercial software systems are supporting connections to or integration with R, such as MATLAB, SAS, etc. |
| **REDCap** | - Marketing is not discussed in case profiling. |

| **Table 11. Summary Comparison of Dependency Hygiene of Academic OSS** | |
| --- | --- |
| **Software tool** | **Characteristics** |
| **3D Slicer** | - Compatibility of the dependencies is considered and checked by means of automated testing. - No established practice of checking potential security issues of dependencies. - The entire 3D Slicer code base is built from source every night on Windows, macOS, and Linux; build results are posted to a public dashboard that is cross-linked to the corresponding source code repositories. |
| **Bioconductor** | - Dependencies on other R packages are from either Bioconductor or CRAN. - Packages rarely depend on third-party software, or software written in other languages. - Deeper dependency graph arises in part from emphasis on re-use of robust and interoperable core- developed infrastructure. - Some packages try to capture A-to-Z ‘workflows’ and thus tend to have many dependencies, these tend to be fragile. |
| **Cytoscape** | - Functions rely on code deployed as services available on web servers. - Generally, such services are callable by Cytoscape or directly by non-Cytoscape clients (e.g., Python) in the larger bioinformatics community. - Some services are provided by other organizations and located in or rely on other GitHub repositories. - Known external repositories containing services that are called by Cytoscape include CXMate, Diffusio. |
| **Globus** | - Transition to a SaaS model has greatly reduced software dependencies. - Only Python libraries for Globus Connect Server, and OpenSSL and SSH for Globus Connect Personal, are included in shipped software, which are generally updated with each software release. - GitHub alerts are received for any security issues for dependent packages. - Dependencies that are not included in Globus software packages are documented in installation instructions. |
| **i2b2 tranSMART** | - Installation guide lists dependencies. - Docker containers are available for easy installation. - Each release is analyzed for dependencies, and to ensure that there is a definitive list of components within the system. |
| **ITK** | - A name-mangling scheme is preventing third- party packages from having name conflicts. - It is easy for users to build. - It does not have a built-in package manager, such as pip for Python. |
| **Linux** | - The Linux kernel has a few dependencies, which are publicly listed. |
| **OHDSI** | - OHDSI depends on individual tools. Some uses R. - WebAPI uses Maven for dependency management. |
| **R** | - Package is self-contained in general. - Dependencies rely on the contributions from its community of developer libraries within the central CRAN. - Distribution is under the terms of the Free Software Foundation’s GNU - Package dependencies are clearly document within the package summary documentation within the CRAN site. |
| **REDCap** | - Web server with PHP, Apache (any OS) or Microsoft IIS (Windows) - MySQL database server - MySQL client - SMTP email server |

# Open Source Software Sustainability Models (OSSSM): Use Cases

## **Use Case: 3D Slicer**

https://slicer.org

**Author of Use Case**: Andrey Fedorov (BWH); Contributors: Steve Pieper, Jean-Christophe Fillion-Robin, Andras Lasso.

**Short History of Tool**: 3D Slicer is a free open-source extensible platform for medical image computing and visualization. Slicer started as a software tool to support planning and visualization for image-guided neurosurgery developed by David Gering as part of his Master’s work at MIT (Gering et al. 1999; Fedorov et al. 2012). Since 1999, Slicer has been under continuous development at the SPL under the leadership of Ron Kikinis. Today it is developed mostly by professional engineers in close collaboration with algorithm developers and application domain scientists, with the participation of Isomics Inc., Kitware Inc., GE Global Research and Queen’s University, and with significant contributions from the growing Slicer community. Initially envisioned as a neurosurgical guidance, visualization and analysis system, over the last decade, Slicer has evolved into an integrated platform that has been applied in a variety of clinical and preclinical research applications as well as for the analysis of non-medical images (Fedorov et al. 2012) as well as a more recent and dynamically updated list of use cases and users here: <https://www.slicer.org/wiki/Main_Page/SlicerCommunity>).

**Who or what is 3D Slicer competition?**

3D Slicer is a standalone desktop application that targets primarily medical imaging researchers and generally anyone with the needs related to volumetric image visualization, analysis, and development of customized application interfaces. For the analysis with the tools that provide similar functionality the reader is referred to (Fedorov et al. 2012).

**Governance:** The source code is hosted under open GitHub organization: <https://github.com/Slicer>. Contribution guidelines are formalized in publicly available document: <https://github.com/Slicer/Slicer/blob/master/CONTRIBUTING.md>, which includes pointers to the developer documentation, formalized decision making process and governance structure. Slicer forum is open for anyone interested in participating, with archives publicly available and searchable: [https://discourse.slicer.org](https://discourse.slicer.org/). Open video conferencing meetings are taking place weekly, with anyone welcomed to join to ask questions or discuss related topics: <https://discourse.slicer.org/c/community/hangout>. Bug tracker is available publicly: <https://issues.slicer.org/>.

Slicer core developers and users meet in person twice a year (NA-MIC Project weeks/hackathons) to work on selected software development topics (application of the platform for specific research and development projects, feasibility studies, integration tests, etc.), provide training, have discussions, and make strategic decisions. These events have been continuously running since 2005, with typical attendance of 40-80 people, at various locations around the world: [https://na- mic.github.io/ProjectWeek/](https://na-mic.github.io/ProjectWeek/). There are several sub-communities, each gathered around a specific Slicer extension (SlicerIGT, SlicerRT, SlicerSALT, SlicerHeart, etc.), with their own regular meetings, repositories, issue trackers, etc.

Decision making is typically happening on the GitHub pull requests, user/developer hangouts and in- person meetings. Decisions are publicly documented via GitHub, forum discussions and Wiki pages. Feasibility studies, experimental designs are documented on “Labs” pages (https://[www.slicer.org/wiki/Documentation/Labs).](http://www.slicer.org/wiki/Documentation/Labs))

As illustrated by the history of pull requests associated with the GitHub project hosting the source code ([https://github.com/Slicer/Slicer/pulls?q=is%3Apr+is%3Aclosed](https://github.com/Slicer/Slicer/pulls?q=is%3Apr%2Bis%3Aclosed), 1066 total as of writing this), there are numerous developers contributing to the development of 3D Slicer. About 10 developers have permission to make modifications to the Slicer core directly, thus it is not relying on a single person. Most extensions are hosted on GitHub and maintained by small developer groups.

Roadmap for the development is available publicly and documented: <https://issues.slicer.org/roadmap_page.php>

Slicer forum, weekly video conferences, GitHub community, in-person bi-annual meetings, training courses organized at major conferences and educational institutions aim to create a diverse and welcoming community.

**Documentation:** Various sources of documentation are available, including Wiki, ReadTheDocs, crowd-sourced documentation, various recipes and YouTube videos. A challenge in managing documentation consistently is due to the extended history of the project (1999 was before GitHub and YouTube!)

Documentation has been provided originally using Wiki (both for users and developers): <https://www.slicer.org/wiki/Documentation/Nightly>. A relatively new effort is to migrate this documentation to the more modern and more usable ReadTheDocs platform: <https://slicer.readthedocs.io/en/latest/index.html>. API documentation is available at <http://apidocs.slicer.org/>.

Tutorials page with slides and sample datasets materials are available <https://www.slicer.org/wiki/Documentation/Nightly/Training>. Non-curated videos on YouTube educating on the use of Slicer: [https://www.youtube.com/results?search_query=3d+slicer&sm=3](https://www.youtube.com/results?search_query=3d%2Bslicer&amp;sm=3)

Commit style guidelines are documented publicly: <https://www.slicer.org/wiki/Documentation/Nightly/Developers/Style_Guide#Commits>, and are consistent (at least, for the most part) with the [Chris’ great guidance](https://chris.beams.io/posts/git-commit/) (see commit history is available https://github.com/Slicer/Slicer/commits/master).

Human-focused readable release notes are posted in the dedicated section of the Slicer forum: <https://discourse.slicer.org/c/announcements/release-notes>

Slicer funded efforts do not have support for internationalization of the documentation, but there are user communities that developed documentation in their languages independently, e.g., [in Chinese](https://discourse.slicer.org/t/slicer-resources-in-chinese/2077).

Scientific publications and other work based on 3D Slicer for over a decade is collected on a Wiki as a reference for people to use when carrying out their use of 3D Slicer: <https://www.slicer.org/wiki/Main_Page/SlicerCommunity/2019>

**Code Quality:** The project does have code styling guidelines documented publicly: <https://www.slicer.org/wiki/Documentation/Nightly/Developers/Style_Guide>

New functionality can be contributed by modifying the core application, or by contributing an extension (similar to applications available in a mobile platforms app stores) with the latter approach being recommended and most practical. Code style guidelines are not enforced or verified for the extensions, delegating that responsibility to the extension contributors.

The project does have testing integrated, as of writing, there is a total of around 700 tests for the core application. Tests for the extensions are managed by the extension contributors. Testing results are available on a public dashboard: <http://slicer.cdash.org/index.php?project=SlicerPreview>.

Contribution process is documented publicly in <https://github.com/Slicer/Slicer/blob/master/CONTRIBUTING.md>. Code review process is enabled by GitHub pull requests. Pull requests are tested using continuous integration.

Entry on BlackDuck/OpenHub with additional metrics: <https://www.openhub.net/p/slicer>

**Support:** Support is provided by the community of developers and users. Only a small fraction of the community has resources for 3D Slicer development. Even smaller fraction (if anyone at all) has dedicated funding for providing support. The main source of support is [https://discourse.slicer.org](https://discourse.slicer.org/)

In spite of the voluntary nature of the 3D Slicer support forum activity, for the over 13,000 forum posts in 2018 the average response time was less than 2 days (or less than 8 hours during weekdays).

Community members are active on several forums and mailing lists where potential users and developers participate (for example, ITK, VTK, CTK forums) and monitor/provide support via various social media platforms (Twitter, ResearchGate, YouTube, Quora, etc.).

**Ecosystem Collaboration:** 3D Slicer is leveraging the ecosystem of related open source projects, which most notably include: VTK, ITK, Qt, CMake, Python, DCMTK. A more extensive list of dependencies is available at <https://github.com/Slicer/Slicer/tree/master/SuperBuild>. Automated testing on all major platforms is utilized to minimize the possibility of regressions and conflicts.

Slicer developers interact on a regular basis with developers for most of the upstream dependencies and actively contribute bug fixes and feature improvements to the appropriate repositories in preference to making local forks.

**Security:** Release binary packages are signed. Most online resources are available via HTTPS. Downloads of the binaries are available via HTTPS. There is no policy to enforce two-factor authentication for contributors. Binary packages are built on highly secured workstations, managed by Kitware staff, protected by strict multi-factor authentication. There is no specific consideration for addressing security aspects, considering the nature of the project.

**Legal:** The project is made available under a non-restrictive (commercial use permitted) license, , however, it is not an OSI-approved license. The specific license used was defined via coordination with the BWH legal department, primarily aiming to mitigate liability risks. License is available as part of the source code repository: <https://github.com/Slicer/Slicer/blob/master/License.txt>

**Finance:** There were several major milestones in the timeline of the 3D Slicer development. The project started as a MS thesis of an MIT student working with clinicians and researchers at Brigham and Women's Hospital (BWH) and Harvard Medical School. In 2001 a commercial company, Isomics Inc, started to support its development and maintenance in close collaboration with BWH and MIT under NIH funding for neuroimaging and neurosurgery research. Two of the core software building blocks of 3D Slicer, VTK and ITK, developed through close collaborations between GE Corporate Research and Kitware, Inc. Over time, developers from both organizations became key contributors as participants with MIT, BWH, and Isomics in the National Alliance for Medical Image Computing (NA-MIC) an NIH project that began in 2004 and lasted for over a decade involving dozens of medical research institutions.  As the official application software of NA-MIC, 3D Slicer was extensively enhanced, modernized, and debugged. Several Canadian research projects have supported major contributions from researchers at Queen's University and the University of Western Ontario.  Many of current research projects are focused on clinical applications of 3D Slicer, but developers also help maintain the core application while customizing it for specific uses. Throughout the lifetime of the project, numerous research grants (primarily NIH) directly or indirectly supported development of 3D Slicer (nearly 50, as of writing). Documented list is available here: <https://www.slicer.org/wiki/Documentation/4.x/Acknowledgments>.

In parallel with the academic sources of support, 3D Slicer continues to benefit from commercially supported development activities. Examples of such activities we can disclose include work with XStrahl [6], SonoVol[7], Rapid LLC [8] or Xoran Technologies [9].  The 3D Slicer software license was specifically designed to enable companies to use 3D Slicer in whole or in part for the development of medical products.  This goal was an explicit motivation in the design of NA-MIC and the ongoing 3D Slicer community because only regulated medical devices can become part of routine patient care.  Today, developers at Isomics, Kitware, Pixel Medical, Ebatinca SL, SonoVol, and other companies actively participate in helping support 3D Slicer functionality that is crucial to their clients or products.

Looking ahead, the 3D Slicer community recognizes the need for ongoing maintenance in order to keep a software project of this scale relevant to the scientific community.  Support for a project of the scope and complexity of 3D Slicer would typically involve teams of dozens if not hundreds in a traditional corporate setting.  The open source nature of 3D Slicer lightens the development burden in some ways, as new developers and even users from anywhere can contribute fixes or documentation and participate in testing.  However, some tasks requiring in-depth knowledge of the application and significant time investment are currently infeasible and are not likely to be undertaken until they are either essential for a commercial project or become the specific target of other research support.

**Marketing:** Project has a logo, web site, publications, forum, bug tracker, and GitHub community, which aim to support the community and improve marketing of the project. However, there is no “marketing strategy” or dedicated personnel to support project marketing.

Official announcements of releases and major events are regularly made via Slicer forum (“Announcements” category). Interesting projects, developments, related efforts are posted on Twitter (https://twitter.com/3DSlicerApp). [3D Slicer YouTube channel](https://www.youtube.com/channel/UC11x1iQ7ydSIFYw4L6wveXg) is used to organize (mostly, community-contributed) educational videos about Slicer.

Small-scale surveys occasionally conducted on the Slicer forum to get general feedback from the community. Information is also collected from the community via feedback forms and during training courses and thematic discussions during project weeks.

**Dependency Hygiene:** There is no established practice of checking potential security issues. Compatibilities are checked by means of automated testing. The entire 3D Slicer code base is built from source every night on Windows, macOS, and Linux. Build results are posted to a public dashboard that is cross-linked to the corresponding source code repositories.

**References**

1. [Fedorov, A., Beichel, R., Kalpathy-Cramer, J., Finet, J., Fillion-Robin, J. C., Pujol, S., Bauer, C., Jennings,](http://paperpile.com/b/hQzOhD/ndfL) [D., Fennessy, F., Sonka, M., Buatti, J., Aylward, S., Miller, J. V., Pieper, S. & Kikinis, R. 3D Slicer as an](http://paperpile.com/b/hQzOhD/ndfL) [image computing platform for the Quantitative Imaging Network.](http://paperpile.com/b/hQzOhD/ndfL) [Magn. Reson. Imaging](http://paperpile.com/b/hQzOhD/ndfL) [30,](http://paperpile.com/b/hQzOhD/ndfL) [1323–1341](http://paperpile.com/b/hQzOhD/ndfL) [(2012).](http://paperpile.com/b/hQzOhD/ndfL)
2. [Gering, D. T. A System for Surgical Planning and Guidance using Image Fusion and Interventional MR.](http://paperpile.com/b/hQzOhD/qwYt) [(1999).](http://paperpile.com/b/hQzOhD/qwYt)
3. [Gering, D., Nabavi, A., Kikinis, R., Hata, N., O’Donnell, L., Grimson, E., Jolesz, F., Black, P. & Wells, W.](http://paperpile.com/b/hQzOhD/5Rxq) [An Integrated Visualization System for Surgical Planning and Guidance using Image Fusion and an Open](http://paperpile.com/b/hQzOhD/5Rxq) MR. J Magn. Reson. Imaging 13, 967-975 (2001).
4. [Kapur, T., Pieper, S., Fedorov, A., Fillion-Robin, J.-C., Halle, M., O’Donnell, L., Lasso, A., Ungi, T., Pinter,](http://paperpile.com/b/hQzOhD/Sad2) [C., Finet, J., Pujol, S., Jagadeesan, J., Tokuda, J., Norton, I., Estepar, R. S. J., Gering, D., Aerts, H. J. W.](http://paperpile.com/b/hQzOhD/Sad2) [L., Jakab, M., Hata, N., Ibanez, L., Blezek, D., Miller, J., Aylward, S., Grimson, W. E. L., Fichtinger, G.,](http://paperpile.com/b/hQzOhD/Sad2) [Wells, W. M., Lorensen, W. E., Schroeder, W. & Kikinis, R. Increasing the impact of medical image](http://paperpile.com/b/hQzOhD/Sad2) [computing using community-based open-access hackathons: The NA-MIC and 3D Slicer experience.](http://paperpile.com/b/hQzOhD/Sad2) [Med. Image Anal.](http://paperpile.com/b/hQzOhD/Sad2) [33,](http://paperpile.com/b/hQzOhD/Sad2) [176–180 (2016).](http://paperpile.com/b/hQzOhD/Sad2)
5. Gering, D.T., Nabavi, A., Kikinis, R., Grimson, W.E.L., Hata, N., Everett, P., Jolesz, F. and Wells, W.M., 1999, September. An integrated visualization system for surgical planning and guidance using image fusion and interventional imaging. In International Conference on Medical Image Computing and Computer-Assisted Intervention (pp. 809-819). Springer, Berlin, Heidelberg.
6. Kitware customer highlight: MuriPlan from Xstrahl – A 3D Slicer based radiotherapy treatment planning system. [*https://blog.kitware.com/kitware-customer-highlight-muriplan-from-xstrahl-a-3d-slicer-based-radiotherapy-treatment-planning-system/*](https://blog.kitware.com/kitware-customer-highlight-muriplan-from-xstrahl-a-3d-slicer-based-radiotherapy-treatment-planning-system/)
7. [*https://blog.kitware.com/kitware-customer-highlight-sonovol/*](https://blog.kitware.com/kitware-customer-highlight-sonovol/)
8. [*https://blog.kitware.com/kitware-customer-highlight-radiopharmaceutical-imaging-and-dosimetry-llc-rapid/*](https://blog.kitware.com/kitware-customer-highlight-radiopharmaceutical-imaging-and-dosimetry-llc-rapid/)
9. [*https://blog.kitware.com/xoran-technologies-and-kitware-collaborate-on-image-guided-platform-for-deep-brain-stimulation-surgery/*](https://blog.kitware.com/xoran-technologies-and-kitware-collaborate-on-image-guided-platform-for-deep-brain-stimulation-surgery/)

## Use Case: Bioconductor

**Author of Use Case**: Guergana Savova, BCH/HMS; Martin Morgan, Roswell Park Comprehensive Cancer Center

**Short History**: Bioconductor provides tools for the analysis and comprehension of high- throughput genomic data. Bioconductor uses the R statistical programming language and is open source and open development. It has two releases each year that follow the semi-annual releases of R. The project started in 2001 at the Dana Farber Cancer Institute. It matured at the Fred Hutchinson Cancer Research Center between 2004 and 2015. The core team has been at Roswell Park Cancer Institute since then.^1-3^

Bioconductor supports many types of high-throughput sequencing data (including DNA, RNA, chromatin immunoprecipitation, Hi-C, methylomes and ribosome profiling) and associated annotation resources; contains mature facilities for microarray analysis; and covers proteomic, metabolomic, flow cytometry, quantitative imaging, cheminformatic and other high-throughput data.^4^

**Governance**: A Technical Advisory Board of key participants meets monthly to support the Bioconductor mission by developing strategies to ensure long-term technical suitability of core infrastructure, and to identify and enable funding strategies for long-term viability. A Scientific Advisory Board including external experts provides annual guidance and accountability.

**Documentation**: Bioconductor documentation comes at three levels: workflows that document complete analyses spanning multiple tools; package vignettes that provide a narrative of the intended uses of a particular package, including detailed executable code examples; and function manual pages with precise descriptions of all inputs and outputs together with working examples. In many cases, users ultimately become developers, making their own algorithms and approaches available to others.

**Code quality**: All Bioconductor packages are built nightly on Windows, macOS, and Linux platforms. Packages must build successfully before propagating to public user-facing repositories. A successful build requires that the package pass a suite of tests defined by R. The tests ensure overall package integrity (e.g., a DESCRIPTION file describing author, title, abstract, software dependencies, license, etc.; correct package structure and language syntax, etc.), functionality (e.g., correct example, vignette, and test code evaluation) and integration with current versions of package dependencies.

**Support**: Users navigate Bioconductor through [views](https://bioconductor.org/packages) https://bioconductor.org/packages/release/BiocViews.html#___Software (a directed acyclic graph of terms from a controlled vocabulary) linking to individual ‘landing pages’ (e.g., [DESeq2](https://bioconductor.org/packages/DESeq2) https://bioconductor.org/packages/release/bioc/html/DESeq2.html) providing an overview of the package, installation instructions, usage statistics, etc. A user-oriented StackOverflow- style support site [https://support.bioconductor.org](https://support.bioconductor.org/) is available and active (100’s of visitors per hour; generally fast response, e.g., < 1 day, by package authors or experts). All packages include maintainer email addresses; many include links to, e.g., GitHub issues. Developer support is provided by the [bioc-devel email list-serve](https://stat.ethz.ch/mailman/listinfo/bioc-devel) https://stat.ethz.ch/mailman/listinfo/bioc-devel. A hybrid [community slack](https://bioc-community.herokuapp.com/) https://bioc-community.herokuapp.com/ is proving to be an effective tool for fostering experienced user / developer collaboration.

**Ecosystem collaboration**: There are 1649 packages from the core group and the international community. Packages have been contributed by more than 1000 maintainers. There are >29,000 mentions of “Bioconductor” in the scientific literature, and 500,000 unique IP addresses in 2018.

**Legal**: Almost all Bioconductor packages are licensed in a way that allows use by any entity without permission; a small number (<10?) specify ‘academic only’ use. Figure 1 lists the licenses associated with R packages in Bioconductor. The main license groups are:

- 1. Artistic license v2 (<https://opensource.org/licenses/Artistic-2.0>) which appears commercially friendly
  2. GPL which requires that distribution of any modified code must make the source available
  3. MIT, BSD, CC fall in the group of lax permissive licenses
  4. Other


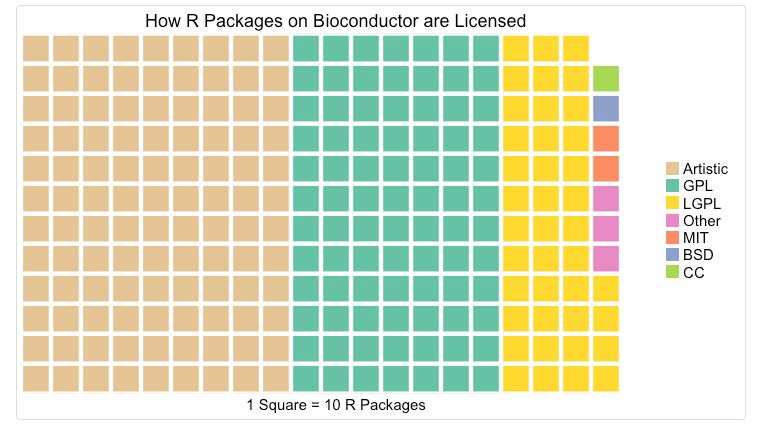
***Figure 1: Bioconductor packages and their licenses (source:*** [***http://seankross.com/2016/08/02/How-R-Packages-are-Licensed.html***](http://seankross.com/2016/08/02/How-R-Packages-are-Licensed.html) ***)***

**Finance**: Initial support was from Institutional funding. NIH / NHGRI support began in 2003 with BISTI grant R33HG002708 and has continued via P41 / U41HG004059 since 2006. NCI / ITCR funding began in 2014 with U24CA180996. Additional grants have been awarded to key stakeholders in the Bioconductor community, with effort directed in part to core project activities; funding sources have included the NSF, European Union, NCI ITCR, NHGRI, and the Silicon Valley Foundation. Details are available in annual reports^4^; these funding sources have not been pursued in a coordinated fashion, but in response to needs and interests of the community. Many individual packages represent the product of federally funded researchers outside the core team; surprisingly, funding sources are almost never acknowledged in contributed packages.

**Marketing**: Bioconductor primarily relies on provision of current and relevant software resources as the primary marketing approach. Bioconductor has a consistent web presence and annual conferences. Social media (e.g., twitter) and video sharing (YouTube) channels exist but are not a primary means of engaging new users.

**Dependency hygiene**: Most Bioconductor packages have dependencies on other R packages. These packages must come from either Bioconductor or CRAN, repositories with conservative standards and commitment to long-term availability. R packages can have system dependencies, typically operating system libraries. Rarely, packages depend on third-party software, or software written in other languages, e.g., python. Bioconductor packages generally have more R package dependencies than non-Bioconductor packages. This deeper dependency graph arises in part from emphasis on re-use of robust and interoperable core- developed infrastructure. Some contributed packages try to capture A-to-Z ‘workflows’; these tend to have a large number of dependencies, are fragile and, perhaps ironically, have limited audiences.

**References**

1. <https://www.bioconductor.org/>
2. <https://en.wikipedia.org/wiki/Bioconductor>
3. Annual reports at <https://bioconductor.org/about/annual-reports>
4. Huber, W. et al. Orchestrating high-throughput genomic analysis with Bioconductor. Nat. Methods 12, 115–121 (2015).

## Use Case: Cytoscape

**Author of Use Case:** Juli Klemm

**Short History of the Tool:** Cytoscape was originally created at the Institute of Systems Biology in Seattle in 2002. Now, it is developed by an international consortium of open source developers. Cytoscape was initially made public in July 2002 (v0.8); the second release (v0.9) was in November 2002, and v1.0 was released in March 2003. Version 1.1.1 is the last stable release for the 1.0 series. Version 2.0 was initially released in 2004; Cytoscape 2.83, the final 2.xx version, was released in May 2012. Version 3.0 was released Feb 1, 2013, and the latest version, 3.7.0, was released in October 2018.

**Governance:** Cytoscape is architected as Core software (maintained by the Core team) augmented by Cytoscape Apps (contributed by community members. The Cytoscape apps are mechanisms for defining and delivering novel calculations and workflows and are available through the Cytoscape App Store, <http://apps.cytoscape.org/>. The Core Team are developers who have GitHub commit access to the Core software.

Cytoscape 3.0 has a clearly defined, simplified API. API jars are strictly separate from the implementation jars. The API is versioned using the [Semantic Versioning standard](http://www.semver.org). This means that the API won't change throughout 3.x, so an app designed to work with an early version of 3.x will be guaranteed to work up to version 4.0 of Cytoscape. Cytoscape maintains an explicit backwards compatibility contract found in each class in the public API so that both core developers and app writers will understand how a class might change. <https://github.com/cytoscape/cytoscape/wiki/Cytoscape-3.0-App-Development>

**Documentation:** Cytoscape has extensive user and developer documentation. The developer resources (<https://cytoscape.org/documentation_developers.html>) include an issue tracker, Nexus repository, nightly builds, and code metrics. In addition, there is a Cytoscape “App Ladder” that teaches essential skills for developing Cytoscape Apps, https://github.com/cytoscape/cytoscape/wiki/Cytoscape-App-Ladder.

User resources include basic and advanced tutorials, a YouTube channel, a blog of published figures, as well as several presentations all available at<https://cytoscape.org/documentation_users.html>

**Code Quality:** Cytoscape core developers use Jenkins, and open source automation tool to support Continuous Integration. It is used to build and test software projects continuously to make it easier for developers to integrate changes to a project. <http://code.cytoscape.org/jenkins/>

Regarding apps, contributors are strongly encouraged to thoroughly test apps before releasing them. However, the Cytoscape team does not independently review them.

**Support:** Cytoscape Help Desk: [https://groups.google.com/forum/#!forum/cytoscape-helpdesk](https://groups.google.com/forum/%23!forum/cytoscape-helpdesk)

**Ecosystem Collaboration:** Cytoscape has an active and diverse community of developers and users. Communication happens mostly via mailing lists and social media:

- The [App Developer Mailing List](https://groups.google.com/forum/%23!forum/cytoscape-app-dev) is for asking questions about app development.
- The [Announcement Mailing List](https://groups.google.com/forum/%23!forum/cytoscape-announce) is used for announcements from the core development team to the rest of the community.
- The [Help Desk](https://groups.google.com/forum/%23!forum/cytoscape-helpdesk) is where users can ask questions on usage.
- [Cytoscape Twitter](https://twitter.com/cytoscape).
- The [Cytoscape Publications Tumblr](http://cytoscape-publications.tumblr.com/) highlights published research using Cytoscape and published plugins.

**Security Legal:** Cytoscape is available as a platform-independent open-source Java application, released under the terms of the [LGPL](http://www.gnu.org/copyleft/lesser.html). By downloading Cytoscape, you agree that you have read the license agreement that follows and agree to its terms. If you do not agree, do not download Cytoscape.

**Finance:** Funding for continued development and maintenance of Cytoscape is provided by the U.S. [National](http://www.nigms.nih.gov/) [Institute of General Medical Sciences (NIGMS)](http://www.nigms.nih.gov/) under award number R01 GM070743. Cytoscape user support, education and new initiatives are supported by the [National Resource for Network](http://nrnb.org/) [Biology (NRNB)](http://nrnb.org/) under award number P41 GM103504.

Cytoscape consortium [http://169.228.38.215](http://169.228.38.215/)

The Cytoscape Consortium holds the intellectual property rights of the following software:

- Cytoscape (LGPL) – [www.cytoscape.org](http://www.cytoscape.org/)
- Cytoscape.js (LGPL) – <http://cytoscape.github.io/cytoscape.js/>NeXO (BSD or similar, in progress) – [http://nexontology.org](http://nexontology.org/)/
- NDEx (BSD or similar, in progress) – [www.ndexbio.org](http://www.ndexbio.org/)

**Marketing:** Cytoscape is marketed through Twitter, Tumblr, Vimeo, and Pinterest as well as mailing lists and forums for specialized audiences.

**Dependency Hygiene:** Note that some Cytoscape functions rely on code deployed as services available on web servers. Generally, such services are callable by Cytoscape or directly by non- Cytoscape clients (e.g., Python) in the larger bioinformatics community. Some services are provided by other organizations (e.g., PSICQUIC for importing public networks), while others are provided by Cytoscape developers (e.g., Diffusion) and are located in or rely on other GitHub repositories. Here is a list of known external repositories containing services called by Cytoscape and maintained by Cytoscape core developers:

- [CXMate](https://github.com/cxmate/cxmate) - adapters that simplify service writing https://github.com/cxmate/cxmate
- [Diffusion](https://github.com/idekerlab/heat-diffusion) - called by Diffusion core app https://github.com/idekerlab/heat-diffusion

Each repo contains information on how to build and deploy the service.

## Use Case: Globus

<https://www.globus.org/>

**Author of Use Case**: Silverstein JC (U Pitt), Davis M (U Pitt), Espino J (U Pitt), Ian Foster (U of Chicago), Gilbertson J (U Pitt), Raumann B (U of Chicago), Taylor D (U Pitt), Zelnis J (U Pitt) and Becich MJ (U Pitt).

**Short History of Tool**: The first public Globus product was the Globus Toolkit: software for enabling distributed computing. The Globus Toolkit was conceived to remove obstacles that prevent seamless collaboration by allowing users to access remote resources while simultaneously preserving local control over who can use resources and when. Foster et. al. continued to develop the Globus Toolkit for fifteen years, trying out several sustainability strategies. The initial releases in 1998 and 1999 were open source. In 2003, an international research consortium, the Globus Alliance,^1^ was formed with the hopes of encouraging broader research and academic support. In 2004, the company Univa^2^ was launched with the hopes of raising money by offering support services for enterprises. In 2005, an industry organization, the Globus Consortium,^3^ with members including IBM, Sun, Hewlett-Packard, and Intel, was formed.

Despite these efforts, the pressure to sustain the Globus mission remained. First, contributors to the Toolkit code were sparse. Very few software developers had the freedom to focus on research productivity at large, which is the core mission of Globus. Contributors were seldom able to provide ongoing maintenance or user support for code they contributed, much less for the rest of the Toolkit. Yet Globus recognized that software sustainability requires continuous updates, adaptations for changing technologies, responsive user support, and enthusiastic users. Second, copies of the code (“forks”) maintained by separate teams meant not all features were accessible to the entire community. Third, requirements and use cases from the research community did not always align with industry requirements and use cases.

In 2009, a new model was adopted with three key elements: a tighter focus on data management for researchers, a “freemium” support model,^4^ and cloud-based Software-as-a-Service (SaaS) delivery.^5^ In 2010 the new service was launched, which was now known simply as Globus. SaaS delivery allowed features needed by researchers that the software toolkit could not provide, such as 24/7 availability, little or no installation or configuration, and simple, intuitive web interfaces, and control of (and responsibility for) the end-to-end user experience.^6,7^ The sustainability strategy was complicated by the fact that the primary users of the SaaS service were researchers (as opposed to Globus Toolkit user who were resource providers and application developers). There were far too many researchers to charge individually, and in any case, researchers were funded by highly competitive grants, so little was available for services like Globus. On the other hand, leading research institutions viewed computing as a strategic investment, and institutional computing centers were charged with acquiring and providing leading-edge services to their researchers. Globus provided benefits research computing organizations recognized, and for which they were already budgeting. Globus realized that while users were researchers, supporters could be institutions, specifically computing resource providers funded to support an institution’s research mission. With this model, the primary software product had to focus on the needs of researchers and the revenue mechanisms had to focus on value for resource providers.

Under the current sustainability model, Globus services are free for researchers while institutions pay an annual subscription fee for access to premium features. Premium features are designed to offer value to research computing directors and, by extension, to the research community they serve. The first subscription was signed in 2013, and, in 2019, the 100th subscriber was signed. Nonetheless, Globus subscribers still only represent a small fraction of those institutions that use Globus worldwide. The freemium approach is essential to the goal of sustaining Globus as a non-profit service for the non-profit research community. By thus operating Globus as a low-cost, “freemium” cloud service, the longevity is ensured of what has become a vital infrastructure component for many resource owners, HPC facilities, and researchers. Globus is operated as a non-profit service within the University of Chicago in order to remain focused on addressing the unique needs of the non-profit research community.

The research community is characterized by many small teams with specialized needs that require custom applications. Research application and service developers establish a growing community that is clearly important to the future of research. These developers need a platform that enables large-scale and automated data management. Although the core Globus product is now aimed at researchers, the Globus mission encompasses support research application and service developers. The Globus service is built on an emerging Platform-as-a-Service (PaaS)^8^: a collection of services with REST APIs,^9^ designed for research application developers, and accessible via a software development kit (SDK) and a command-line interface (CLI). Globus SaaS itself uses the public Globus PaaS for building and operating the primary Globus service. Substantial investment is planned in expanding the platform’s functionality so that developers can build richer solutions for their research communities.^10^

**Governance:** The Globus project is governed by its PI, Ian Foster, and the University of Chicago. The Globus SaaS is operated out of the University of Chicago as a service core to the non-profit research community. Technical, business, and management decisions are the responsibility of Globus staff. Institutional subscribers via subscriber meetings and consultations, and community users via mail lists, webinars and on-site tutorials, provide input on feature prioritization, product roadmap, subscription pricing, and sustainability models.

**Documentation:** Globus provides fully developed documentation for end-users, developers and administrators. This documentation adequately covers a wide range of topics including guides for installation, FAQs, API usage, tutorials, etc.^11^ Each software release is clearly versioned with release notes and change history documented.

**Code quality**: Globus has a professionally maintained and managed code base that is developed with best practices such as version control and software management in a source code repository (GitHub), extensive documentation and code comments, linting, distribution of knowledge across team members, and incremental/iterative software development. Code is reviewed by at least one other software engineer before release to production. Globus employs a continuous integration environment, automated tests, and documented, standardized human QA testing to ensure code quality. Globus developers follow secure development practices, including OWASP recommendations to prevent web application security risks.

**Support**: Globus provides several support options from online self-help tools, listserv groups to a ticketing submission system with a responsive, dedicated support team.^12^ Globus subscribers are guaranteed a response time of one business day to support tickets.^13^

**Ecosystem collaboration:** Globus has a large user base of over 200,000 registered users and 140 institutional subscribers. Globus is widely used across the physical, social, and life sciences, including at most leading US universities and many sites overseas. Globus moves thousands of terabytes a day of research data, and there are 2,000 active multi-user storage systems (e.g., campus storage systems) and over 20,000 active single user storage systems (e.g., laptops) accessible via Globus. A strong community is supported by multiple channels of user engagement. Daily interaction between users and the Globus team occurs through the robust ticketing system and active list serv groups. Numerous webinars and on-site, two-day workshops occur every year at research institutions across the US.^14^ The multi-day annual user conference provides a forum for users and subscribers to connect with the Globus team as well as with each other. The conference includes a half day meeting for subscribers to provide input into feature priorities, overall product roadmap, subscription pricing, and sustainability models.^15^ The Globus customer engagement team facilitates application of current Globus services by ensuring that institutions provide the maximum value of their subscription to their researchers. The Globus professional services team engages in grant-funded collaborations with research teams to apply, extend, develop, or innovate Globus services in the context of science drivers.

**Security:** Globus has maintained a strong security model for many years, using highly standards-based components and protocols that address message protection, authentication, delegation and authorization for distributed infrastructures. Globus has adopted new technologies and procedures to ensure the continued security of data exchange For example, the evolution from WS-security-compliant message-level and credentials security using X.509 to the current model of OAuth, OpenSSL and other new standards and the increased security posture through adoption of more rigorous security controls.

Globus services leverage federated login and allow user authentication using one of the many supported identity providers (e.g., institutional identities, eRA Commons, ORCID, Google). Since Globus acts as an identity broker and uses federated login, institutional credentials are never seen by Globus. Globus Auth, the identity and access management service in Globus, is based on OAuth 2 and is used to secure all Globus services and used for integration with third-party applications. This provides an advanced, user consent based delegated authorization model that allow applications and services to act on behalf of users and other services.

Globus uses a “data channel” for moving data between two endpoints. This data channel is established directly between the source and destination endpoints and cannot be accessed by the Globus service, only by the servers running on the endpoints. Transfers can be encrypted using OpenSSL libraries installed at the endpoint and TLS 1.2. Transfer of designated restricted data are always encrypted. In addition to the data channel, Globus uses a "control channel" to communicate with the source and destination endpoints for a transfer. The control channel is encrypted with TLS 1.2 or higher.

In 2017, the Globus introduced a high assurance tier that provides additional security controls to meet the higher authentication and authorization standards required for access to restricted data, such as Protected Health Information, Personally Identiﬁable Information, and Controlled Unclassiﬁed Information. Users must authenticate with speciﬁc identities as determined by the policy set by administrators at the institution to obtain access. In addition, users must re-authenticate in each new application session with the required identity and each authentication lasts for a speciﬁc period of time after which the user must re-authenticate. A detailed audit trail is generated that allows reconstruction of data access and user activities.^16^

Globus has undergone security reviews by various external bodies, including source code reviews by the NSF Trusted CI center and HIPAA risk and gap analyses by third party auditors.

**Legal:** The Globus legal approach to software distribution has evolved with the Globus technology. Currently Globus uses a variety of licensing models. Select software components operated as a service and client-side software, such as the Globus Command Line Interface, the Globus Software Development Kit, and some version of Globus Connect (the software that enables storage systems to be accessed via the Globus service) are made available under the Apache 2.0 license. Some versions of Globus Connect are licensed under the Globus Community License, under which subscribers can access source code for the purposes of code review and contribution.^17^ Globus does not license the remaining software components it operates as a service.

Globus holds no patents on Globus technology. Globus has proposed that the University of Chicago enter into a software code escrow agreement with its federal funding agencies that would provide all Globus source code and related materials be transferred to the research community if the Globus team is no longer willing or able to provide the Globus service to the non-profit research community. Globus would transfer all the relevant architecture documents and artifacts (including source code, design documents, test plans, files, working plans, database designs etc. when applicable) for all information systems and provide assistance and open communications until the transfer were complete.

**Finance:** To enable long-term sustainability and alignment with science needs, Globus is provided to the community via a freemium model. Data transfer is free; other services (e.g., data sharing, usage management services for research computing administrators) are provided under an annual subscription by the institution. This subscription model is designed to allow research institutions to engage with Globus as subscribers, and then make premium Globus services freely available to their researchers, educators, and students. Furthermore, the basic Globus data transfer capability is made freely available to researchers and educators in the non-profit sector. Thus, any collaborator of researchers at a subscribing institution can access data that those researchers make available via Globus. Globus focuses non-profit research use; however, commercial use is allowed.^13^

Subscriptions are a flat annual fee that allow unlimited use. The fee is based on the institution’s level of research activity, in order to ensure equitable pricing. Hence, large research institutions pay more than a regional college with a limited research program. In the US, pricing is determined by the institution’s Carnegie Classification (carnegieclassifications.iu.edu); for non-US institutions, an attempt is made to find a suitable proxy by looking at the number of researchers served and the research budget. Pricing is higher for commercial organizations and subscription levels are structured differently for commercial use (most notably, commercial subscriptions are not unlimited use).

Although under the freemium model an increasing portion of ongoing funding has shifted from federal grants to institutional subscriptions, federal funding is still important for developing new capabilities—which are then sustained via subscriptions.

**Marketing:** Globus attributes their brand recognition primarily to sustained, programmatic outreach via multiple channels, with a set of consistent messages, and a long history of high quality. In addition, Globus brand recognition is strengthened by a memorable, uncomplicated name and a simple logo. Time and resources have been invested into a well-designed public website that is optimized for search.

A variety of communication methods keep Globus visible and the user community updated:

- Speaking engagements and participation in workshops, working groups, and conferences
- Presence on social media (Twitter and LinkedIn, primarily
- Presence in news media via occasional press releases and article postings
- Blog with frequent (ideally one to two per month) announcements and articles
- Email announcements to opt-in marketing database that include monthly newsletters and periodic announcements for new blogs, news releases and events
- Email announcements to user and admin email lists maintained
- Engagement with partners (e.g. via joint webinars, event sponsorship, etc.)
- Workshops and user conferences, for example the annual Globus users conference and on-site tutorials and workshops.

**Dependency Hygiene:** Most, if not all, software has some dependencies on other software or libraries, Globus is no exception. However, the transition to a SaaS model has greatly reduced the software dependencies. Only Python libraries for Globus Connect Server and OpenSSL and SSH for Globus Connect Personal are included in shipped software. These dependencies are generally updated with each software release. During installation, the user is prompted to install dependencies that are not included in Globus software packages. Updates of these dependencies is the responsibility of the storage administrator, who can get the list of these dependencies by querying the Globus software packages.

**References**

1. Press release: Globus Alliance established as international consortium to advance Globus grid software. Sept. 2, 2003. (http://toolkit.globus.org/alliance/news/prGAannounce.html)

2. Jennifer Mears. 10 start-ups to watch: Univa. Network World, April 25, 2005. (https://www.networkworld.com/article/2320321/10-start-ups-to-watch--univa.html)

3. Nicole Hemsoth. HP, IBM, Intel, Sun launch Globus Consortium, HPCwire, January 31, 2005. (https://www.hpcwire.com/2005/01/31/hp_ibm_intel_sun_launch_globus_consortium/

4. https://en.wikipedia.org/wiki/Freemium

5. https://en.wikipedia.org/wiki/Software_as_a_service

6. L. Childers, L. Liming, and I. Foster. Perspectives on Distributed Computing: Thirty People, Four User Types, and the Distributed Computing User Experience. Argonne National Laboratory Technical Report ANL/MCS/CI-31, September 2008.

7. “Software-as-a-Service as a path to software sustainability” by Foster, Vasiladis and Tuecke https://www.globus.org/sites/default/files/saas-as-a-path-to-sustainable-software-delivery.pdf

8. https://en.wikipedia.org/wiki/Platform_as_a_service

9. https://www.codecademy.com/articles/what-is-rest

10. Liming, Lee, & Vasiliadis, Vas. “Globus and the Path to Sustainability.” Science Gateways Community Institute (blog), May 9, 2019, https://sciencegateways.org/-/guest-blog-globus-and-the-path-to-sustainability

11. https://docs.globus.org/

12. https://www.globus.org/contact- us

13. https://www.globus.org/subscriptions

14. https://www.globus.org/events.

15. https://www.globusworld.org/

16. <https://docs.globus.org/security/>

17. https://www.globus.org/legal/software-license

## Use Case: i2b2 tranSMART

**Author of Use Case:** Jonathan Silverstein (U Pitt), Michael J. Becich (U Pitt), Keith Elliston (tranSMART Foundation & Axiomedix, Inc.), and Ye Ye (U Pitt)

**Short History of Tool** The i2b2 project was established in 2004 as an NIH-funded National Center for Biomedical Computing at Brigham and Women’s Hospital and was later based at Partners HealthCare System. The i2b2 Foundation was formed in 2016, to provide oversight and governance for the i2b2 project. The i2b2 platform develops a web-enabled warehouse server and software tools, enabling researchers to retrieve patient cohorts and obtain project-specific databases (integration of medical record and clinical research data) while preserving patient privacy.^1^ This platform also provides a set of web services to load additional data, such as applying natural language processing service to determine patients’ smoking history from discharge summaries.^2^ Over 250 hospitals and research centers have adopted the i2b2 platform for sharing, integration, standardization, and analysis of heterogeneous data from healthcare and research. i2b2 facilitates three different data integration approaches: one based on ontology, one based on the tranSMART engine, and the third based on CouchDB.^3^

The tranSMART Foundation was established in 2013 as a public-private partnership between scientists in the United States and the European Union. Founding partners include the University of Michigan, the Pistoia Alliance and Imperial College London. The initial version of tranSMART’s data management system was developed in 2009 by scientists at Johnson & Johnson and Recombinant Data Corporation, and mainly used by pharmaceutical researchers. TranSMART has being developed to support one informatics-based analysis and pre-competitive data-sharing platform for clinical and translational research,^4^ enabling hypothesis generation and validation using integrated data.^5^ The development currently focuses on TraIT and eTRIKS, which are based on the tranSMART platform, supported by major European translational research initiatives.

In May 2017, the i2b2 Foundation (Informatics for Integrating Biology and the Bedside) and the tranSMART Foundation merged to create a single, unified organization, i2b2 tranSMART Foundation to advance the field of precision medicine. In 2019, the i2b2 tranSMART Foundation worked with Partners Healthcare to release i2b2 under the weakly permissive MPL2 open source license (previously i2b2 was distributed under the BWH License, with was not Open Source Definition compliant).

**Governance:** The governance structure of the i2b2 tranSMART Foundation is defined in their Bylaws available publicly <https://drive.google.com/file/d/0B8lizkKDeaKhTUF3QmNTTFk0ZnM/view>. The governance structure makes the foundation not rely on any single person. High-level leaders include board of directors, executive committee, governance/nominations committee, finance/audit committee, and technology committee. The membership program includes stakeholders based on merit and contribution, and this group is actively engaged in operations, fundraising, software development, and the nomination and election of new board members. In addition, members participate in working groups, such as ETL working group, ontology working group, and user interfaces working group.

Each of the software projects organized by the i2b2 tranSMART Foundation are governed by Project Management Committees (PMCs) that are modeled after the “Apache Way.” These PMCs provide coordination of development, establish standards for testing, and manage the release process of the platforms.

The i2b2 tranSMART foundation provides many ways to involve new users and contributions, including free training class, working groups open to anyone, sign-up mailing list, sponsorship programs, user platforms, and vendor sign up link. Monthly webinars have been organizing since March 20, 2018, (and since 2014 under the predecessor tranSMART Foundation) during each of which a community member presents an update on the foundation and a preview of upcoming events. These recorded webinars are publicly available online through YouTube <https://www.youtube.com/channel/UC3hy0Az4VYs6TZgN79-w8TA>.

**Documentation:** The i2b2 community provides updated documentation through a Wiki page <https://community.i2b2.org/wiki/pages/viewpage.action?pageId=342684>. The documentation is organized for different interest groups. For beginners, documentation includes installation guide, upgrade guide, and tutorials. For developers, documentation includes server-side messaging, server architecture, server-side design, web client design, and release notes. For end users, documentation includes web client help and workbench user guide. The i2b2 documentation lists changes of each release and bug fixes. For each bug fix, i2b2 documentation provides a bug ID, a one sentence summary, and details about the content and reasons of changes and affected versions. Commit messages in the i2b2 GitHub <https://github.com/i2b2> are simpler, listing changes in codes. The i2b2 provides human-focused release notes for every release.

The tranSMART community provides documentation on its current version 16.3. The documentation includes instructions on different functions: browse, analyze, summary statistics, advanced analyses and visualizations, export results, sample explorer, gene signature wizard, and genome-wide association study tool. The tranSMART GitHub website <https://github.com/transmart> includes commit messages for each change of codes.

**Code Quality:** Both i2b2 and tranSMART have extensive automated and manual testing as a part of their well define release process. Example test plans for i2b2 include:

<https://community.i2b2.org/wiki/display/HOM/HOM+Home?preview=%2F336164%2F336376%2FUETL+Test+Plan.docx>

**Support:** The i2b2 foundation has established an i2b2 Bug Tracker for contributors to receive suggestions for improvements. <https://community.i2b2.org/jira/secure/Dashboard.jspa>. Outside users can sign an account to review the records of historical and current issues and solutions. The i2b2 Bug Tracker system flags important issues as *ToDo*, *In Progress*, *Done*, and *Other Issues*. It also allows each user to track his/her specific issues. In addition, a publicly available Google Forum is organized to assist installation <https://groups.google.com/forum/#!forum/i2b2-install-help>.

TranSMART has a platform for developers and testers through tranSMART Wiki <https://wiki.transmartfoundation.org/display/transmartwiki/tranSMART+Project+wiki+Home>, which is not currently accessible.

**Ecosystem Collaboration:** The i2b2 tranSMART Foundation provides a platform for contributions of new tools. Guidelines of submitting new contributions are available through <https://transmartfoundation.org/transmart-platform-code-contributions/>.

In addition, the i2b2 platform provides information of finished and ongoing community projects through <https://community.i2b2.org/wiki/display/i2b2/i2b2+Community+Projects>. For example, the i2b2 FHIR Cell project allows i2b2 core to communicate with SMART cells using the Fast Healthcare Interoperability Resources (FHIR).

**Security:** In the i2b2 platform, securable remote access is provided through well-deﬁned messages in web services. Anonymous patients’ aggregate counts obfuscated by adding small random numbers are returned in queries in order to protect patients’ privacy.^1^ Moreover, the i2b2 PM Cell Security Enhancements project consists of modifications to the i2b2 core project management cell in order to increase the security of the authentication information <https://www.i2b2.org/software/contributed.html>. The security enhancement is becoming the focus of the next core release.

The tranSMART platform also has a mechanism to manage security for studies. The protect study operation needs to receive an approval of administrator. After a user loads study data into a database server, tranSMART administrator will assess whether a study is a secure object or not. If a study is deployed on multiple servers, then the authorization must be conducted on each server separately <https://transmart.support.axiomedix.com/hc/en-us/articles/360005847334-Managing-Security-for-Studies>.

**Legal:** The i2b2 and tranSMART are free open source software <https://transmartfoundation.org/legallicensing/>

The i2b2 software is licensed through the Mozilla Public License (MPL) version 2.0 <https://www.mozilla.org/en-US/MPL/2.0/> under the terms of the Healthcare Disclaimer addendum https://community.i2b2.org/wiki/display/webclient/The+i2b2+MPL+2.0+License+with+Healthcare+Disclaimer+Addendum

The tranSMART software is now made available under the terms of the version 3 of the GNU General Public License (GPL v3) <https://www.gnu.org/licenses/gpl-3.0.html>

**Finance:** To help financially support the i2b2 tranSMART operations, the Foundation offers four sponsorship programs, including contributing sponsors (medical and academic research centers and small vendors), corporate sponsors (large corporate supported), sustaining sponsors (organizations providing on-going staff and financial support), event sponsors (vendors who offer product or services). <https://transmartfoundation.org/contributing-sponsors-program/>

- Contributing sponsors: Michigan Medicine, University of Michigan, U of Kansas Medical Center, ITTM (Information Technology for Translational Medicine), Beth Israel Deaconess Medical Center, University Medical Center Göttingen, Prognosis Data, InterSystems, Boston Children's Hospital Computational Health Informatics Program, Wake Forest Clinical and Translational Science Institute
- Corporate sponsors: Takeda
- Sustaining sponsors: Department of Biomedical Informatics, Harvard Medical School, Partners Healthcare, Harvard Catalyst, Axiomedix
- Event sponsors: Department of Biomedical informatics, Harvard University, Harvard Catalyst, Persistent, Essex, Axiomedix

Complementary with the nonprofit sustainability model through the tranSMART and the successor i2b2-tranSMART Foundation efforts, the team that founded the tranSMART and i2b2 tranSMART Foundations, Keith Elliston and colleagues, started Axiomedix in 2018 specifically to offer a four-part commercial support mechanism to sustainably support open source software projects, particularly after their grant funding cycles have ended:

First, Axiomedix offers a commercial grade software publishing and support model. For this, Axiomedix works with developers to create a supportable, tested and validated version of the open-source platform, running on a commercial grade and updated technology stack. So far, Axiomedix supports i2b2 and tranSMART, and is working to support a number of other platforms (Arvados, CWL, openBEL and others). In this model, Axiomedix pays a royalty on all sales to the OSS project (if applicable and possible) and pays a royalty to the developers in return for their work to develop and support the commercial version, providing tier III and IV support through the Axiomedix support portal (support.axiomedix.com).

Second, Axiomedix offers full-service solutions, which include installation, configuration, data loading, curation, training and more. Axiomedix works directly with developers to provide these services, as high-value contract work.

Third, Axiomedix has developed the Axiomedix Expert Network, which is a network of core developers that can perform part-time, contract work with commercial customers on an hourly basis. This enables an efficient development of new capabilities for the platforms, and a direct integration of these enhancements into the core open-source codebase. For all of these efforts, Axiomedix developed close relationships with the core developers of the open-source platforms, which can provide them with sustainable income streams even after the grant funding for these projects has ended.

Fourth, Axiomedix has brought together highly skilled technologists, open source technologies and subject matter experts, to develop new software products that enable core aspects of precision medicine. These projects can be funded by grants, sponsors or investors, and can result in new open source products or commercially developed products. This approach enables the development of new innovations on the core capabilities of the scientific open source ecosystem.

**Marketing:** The i2b2 tranSMART foundation has a logo, website, and its own trademarks. Official announcements are available through <https://transmartfoundation.org/news/>. The foundation has set up many workgroup mail lists. The tranSMART platform has additional system administrator and developer mail lists. Moreover, the foundation has established many communication channels to announce and highlight interesting events and topics:

Twitter: <https://twitter.com/i2b2tranSMART>

LinkedIn: <https://www.linkedin.com/groups/?home=&gid=4218734>

Facebook: <https://www.facebook.com/i2b2tranSMARTfoundation/>

Google forum: <https://groups.google.com/forum/#!forum/transmart-discuss>

YouTube channel: <https://www.youtube.com/channel/UC3hy0Az4VYs6TZgN79-w8TA>

**Dependency Hygiene:** Both the i2b2 workbench developer’s guide and i2b2 installation guide provide information about dependencies. In addition, the i2b2 developers create three Docker contains “i2b2-web,” “i2b2-wildfly,” and “i2b2-pg” to encapsulate the core functionalities and facilitate the configuration of numerous dependencies in i2b2 components.^6^

The TranSMART ETL guide <https://www.etriks.org/wp-content/uploads/2015/12/etl-getting-started-4.pdf> lists dependencies, such as pentaho data-integration software suite version 4.4.0., Java Runtime Environment (JRE), SSH client for secure connection, postgres client to database connection. Each release is analyzed for dependencies, and to ensure that there is a definitive list of components within the system. TranSMART Docker is available at <https://github.com/hms-dbmi/transmart-docker>

**References:**

1. Murphy SN, Weber G, Mendis M, Gainer V, Chueh HC, Churchill S, Kohane I. Serving the enterprise and beyond with informatics for integrating biology and the bedside (i2b2). Journal of the American Medical Informatics Association. 2010 Mar 1;17(2):124-30.
2. Murphy SN, Mendis ME, Berkowitz DA, Kohane I, Chueh HC. Integration of clinical and genetic data in the i2b2 architecture. In AMIA Annual Symposium Proceedings 2006 (Vol. 2006, p. 1040). American Medical Informatics Association.
3. Murphy SN, Avillach P, Bellazzi R, Phillips L, Gabetta M, Eran A, McDuffie MT, Kohane IS. Combining clinical and genomics queries using i2b2–Three methods. PLoS One. 2017 Apr 7;12(4):e0172187.
4. Athey BD, Braxenthaler M, Haas M, Guo Y. tranSMART: an open source and community-driven informatics and data sharing platform for clinical and translational research. AMIA Summits on Translational Science Proceedings. 2013: 6.
5. Scheufele E, Aronzon D, Coopersmith R, McDuffie MT, Kapoor M, Uhrich CA, Avitabile JE, Liu J, Housman D, Palchuk MB. TranSMART: an open source knowledge management and high content data analytics platform. AMIA Summits on Translational Science Proceedings. 2014:96.
6. Wagholikar KB, Dessai P, Sanz J, Mendis ME, Bell DS, Murphy SN. Implementation of informatics for integrating biology and the bedside (i2b2) platform as Docker containers. BMC medical informatics and decision making. 2018 Dec;18(1):66.

## Use Case: ITK

[https://itk.org](https://itk.org/)

**Author of Use Case:** Sarachan BD (GE Research), Miller JV (GE Research), Fedorov A (BWH).

**Short History of Tool:** In 1999 the National Library of Medicine (NLM) and NIH awarded a three-year contract to develop an open-source image registration and segmentation toolkit, which eventually came to be known as the Insight Toolkit (ITK). The images in question are typically from medical images such as CT or MRI scanners. Registration refers to aligning the or developing correspondences between image data. Segmentation refers to identifying and classifying data in the images, such as nodules in lung. An original goal of ITK was to process data from the Visible Human project which included CT, MRI, and cryosections. Since then, it has been applied successfully for the analysis of a broad range of imaging modalities, including non-clinical images, such as electron microscopy. ITK is implemented in C++ and provides a class library for developing software applications involving image registration and segmentation.

The ITK consortium members included three companies: GE Research, Kitware, Inc., and MathSoft (now called Insightful), and three academic members: University of North Carolina, University of Tennessee, and University of Pennsylvania. Each organization had its own PI. The GE PI was Bill Lorensen, a GE Coolidge Fellow, which is the highest honor awarded to GE Research employees. The overall program Project Manager was Dr. Terry Yoo from NLM. Other contributing organizations include Brigham & Women’s Hospital, Columbia University, and University of Pittsburgh. There were many NIH companion grants and contracts that further developed ITK.

ITK has been used in a number of commercial products from GE and likely other companies, ITK has also been used internally to GE to build important internal applications, such as for industrial inspection, even beyond the healthcare industry. GE maintains its own extension to ITK consisting of algorithms that are either proprietary or not generalized enough for community use.

ITK has also been instrumental as a component to numerous end-user medical image computing research tools. Some of the most prominent examples of such software packages include 3D Slicer ([https://slicer.org](https://slicer.org/)) and MITK (<http://mitk.org/>).

Although ITK is a C++ library, there are several packages that provide Python packaging (i.e., SimpleITK and itk-python) further increasing usability of the software and integration with the other widely popular Python tools.

**Who or what is ITK’s competition?**

There are commercial services that provide clinically approved image diagnostics and commercial software and services for developing purpose-specific image analysis algorithms (e.g. Definiens). We are not aware of an open source competitor to ITK having its breadth of capabilities and flexibility specifically for radiology. Packages such as ImageJ are popular for microscopy image viewing and analysis. ITK has been used a component of several other open source software.

**Governance:** A non-profit ITK consortium has been formed that manages ITK licensing, owns the copyright, and acts as a governing body. Code changes are controlled by strictly limiting who has access to commit changes to the repositories. There is a code review process established for submissions. An online ITK journal documents changes and acts as a forum for proposed ITK changes. ITK development is maintained fully via GitHub, which hosts the source code, release packages, and bug tracker (see <https://github.com/InsightSoftwareConsortium/ITK>). Contribution guidelines are documented, and there are numerous contributions from the community to its content.

**Documentation:** There is much inline documentation as well as a user’s guide that has been published as a book. Coding example are automatically built as part of nightly tests. There are strictly- enforced coding conventions such as consistent naming rules.

**Code quality:** ITK had automated nightly builds and tests as far back as 1999, being an early adopter of this software-engineering best practice, before the widespread adoption of continuous integration and GitHub.

**Support:** ITK has mailing lists and over its long history has had dedicated volunteers who would give detailed help to users even including example code. ITK has its own Discourse forum for discussions ([https://discourse.itk.org](https://discourse.itk.org/)) and mutual help among users. Very importantly, NIH has continued to provide maintenance contracts for bug fixes, incremental improvements, and a moderate level of user support (i.e. brief answers to questions). This maintenance has typically been performed by Kitware, providing continuity and expertise. Kitware also offers commercial ITK support for a fee.

**Ecosystem collaboration:** There have been many contributions to ITK over time, including community members not specifically funded for contributing to ITK. As of this writing, there have been over 200 developers contributing to the source code of ITK, according to the automatically collected statistics by GitHub, and available publicly at <https://github.com/InsightSoftwareConsortium/ITK/graphs/contributors>.

**Security:** Security has not been a primary consideration for ITK, which is typically embedded within broader systems. That being said, ITK is likely not particularly vulnerable to cyber-threats. Being implemented in a low-level language (C++) some types of memory hacks may be possible.

**Legal:** Initially ITK was licensed under BSD. This was later changed to Apache which requires contributors to abide by the open source requirements. The Debian operating system distributes ITK and performs scans to ensure there are no embedded third-party packages having license restrictions. Initially ITK permitted the inclusion of patented software, but after the administrative of these special cases became burdensome, patented materials were no longer permitted for inclusion.

**Finance:** As mentioned above, ITK is free to users, NIH has provided continual funding for maintenance, and commercial-grade support can be purchased.

**Marketing:** Initial visibility for ITK was via promotion at medical imaging conferences. Over time, the use of ITK for medical image analysis became pervasive.

**Dependency Hygiene:** ITK has managed its dependencies using a name-mangling scheme that would prevent third- party packages from having name conflicts. This would be somewhat laborious for the ITK developers but resulted in ITK being very easy for users to build. ITK does not have a built-in package manager such as pip for Python.

**References**

1. [https://itk.org](https://itk.org/)
2. <https://en.wikipedia.org/wiki/Insight_Segmentation_and_Registration_Toolkit>
3. <https://www.kitware.com/>
4. <https://www.definiens.com/>

## Use Case: Linux

**Author of Use Case**: Guergana Savova, BCH/HMS

**Short History**: Linux is the world’s largest and most pervasive open source software project in the history of computing. The Linux kernel which was released by Linus Torvalds in 1991 is the largest component of the Linux operating system. Linux has become the world’s most dominant operating system with adoption in various sectors including finance, government, and education. “it is also the operating system of choice to support cutting-edge technologies such as the Internet of Things, cloud computing, and big data.”^1^

**Governance**: “Linux is the premier example of open source sustainability and success. The non-profit Linux Foundation founded in 2000 provides a neutral home where Linux kernel development can be protected and supported for years to come:

- Linux Foundation fellowships sponsor the work of the Linux creator Linus Torvalds and lead maintainer Greg Kroah-Hartman
- Linux Foundation IT operations run the systems behind Linux kernel development on kernel.org
- Linux Foundation Training offers free and paid training courses and Linux certifications
- Linux Foundation Events organize gatherings where kernel developers can collaborate”^1^

The Linux Foundation Board of Directors is comprised of 22 senior leaders from across the IT industry. Board members represent Linux Foundation members and the Linux developer community and set the strategic direction for the organization.

**Documentation**: The Linux kernel provided user’s and administrator’s guides available at <https://www.kernel.org/doc/html/latest/> Code is available at <https://github.com/torvalds/linux>

**Support**: Support is provided through the LF JIRA at <https://support.linuxfoundation.org/>

**Ecosystem collaboration**: “A strong and vibrant community

- 13,594 developers from at least 1,340 companies have contributed to the Linux kernel since 2005
- >1,600 developers contributed to each kernel version
- A new major kernel release occurs every 9 – 10 weeks
- The Linux kernel community merged changes at an average rate of 7.8 patches per hour over the past 15 months.

A thriving commercial ecosystem

- 95%+ of the top 1M web domains
- 80%+ of smartphones run Android (based on the Linux kernel)
- 98%+ of the top 500 fastest supercomputers in the world
- Most of the global markets, including NYSE, NASDAQ, London Exchange, Tokyo Stock Exchange
- The majority of consumer electronic devices
- 75%+ of cloud-enabled enterprises report using Linux as their primary cloud platform
- E-commerce giants Amazon, EBay, PayPal, Walmart, and more run on Linux”^1^

**Legal**: The Linux kernel is released under GNU General Public License (GPL) v2 which allows distribution and sale of possibly modified and unmodified versions but requires that all those copies be released under the same license and be accompanied by the complete corresponding source code.^2^

**Finance**: The Linux Foundation is a membership organization based on different levels of membership.^3^ Individual memberships are $49 per year.^4^ Corporate annual membership has Platinum ($500,000), Gold ($100,000) and Silver ($5,000-$20,000) levels. Silver dues are based on total corporate consolidated headcount of the parent company.^5^ The Linux Foundation has over 1,000 corporate members and thousands of individual supporters.^6^

**Marketing**: As of March 2016, top corporate contributors to Linux kernel include Intel, Red Hat, IBM, Motorola, Linaro, Google, Mellanox, SUSE, AMD, Renesas Electronics, Samsung, Rockchip, Oracle, ARM, Canonical, Broadcom.^7^ The Linux system is available as a product from Red Hat. Red Hat provides storage, operating system platforms, middleware, applications, management products, and support, training, and consulting services.

**Dependency hygiene**: The Linux kernel has a few dependencies listed at this website.^8^

**References**

1. <https://www.linuxfoundation.org/projects/linux/>
2. <https://en.wikipedia.org/wiki/Linux_kernel>
3. <https://www.linuxfoundation.org/membership/>
4. <https://www.linuxfoundation.org/membership/individual/faq/>
5. <https://www.linuxfoundation.org/projects/deep-learning/join/>
6. <https://www.linuxfoundation.org/about/>
7. <https://lwn.net/Articles/736578/>
8. <https://www.kernel.org/doc/html/v4.13/process/changes.html>

## Use Case: OHDSI

<https://www.ohdsi.org/data-standardization/the-common-data-model/>

**Author of Use Case**: GQ Zhang (UTHSC) and Richard Boyce (U Pitt)

**Short History of the Tool:** Observational Health Data Sciences and Informatics (OHDSI) and was initiated in 2013 as a follow-up to the Observational Medical Outcomes Partnership (OMOP). OHDSI is a multi-stakeholder interdisciplinary collaborative that is striving to bring out the value of observational health data through large-scale analytics. The main objective of OHDSI is to establish a research community and open source tool set for observational health data sciences that enables active engagement across multiple disciplines spanning multiple stakeholder groups.

**Governance:** OHDSI has established an international network of researchers and observational health databases with a central coordinating center housed at Columbia University. OHDSI is not a single open source software. Rather, it is a collection of open-source tools to make analysis of observational health data more rapid, rigorous, and reproducible. The OMOP common data model related standard vocabulary are the foundational tools for all OHDSI initiatives: <https://github.com/OHDSI/CommonDataModel>

**Documentation:** [http://www.ohdsi.org/web/wiki/doku.php?id=documentation:overview](http://www.ohdsi.org/web/wiki/doku.php?id=documentation%3Aoverview)

Documentation available about how to get started with OHDSI, Common Data Model (CDM), ETL creation best practices, and Tool Specific Documentation.

**Code Quality:** Varies since a large collection of tools are developed around the OMOP CDM. https://github.com/OHDSI

**Support:** The OHDSI community provides two methods for receiving support. The community-based Discourse forum (forums.ohdsi.org) provides support for implementing OHDSI tools, proposing or participating in network research studies, or requesting information on OHDSI related topics. Technical questions specific to software are managed through issue tickets that anyone can open on the various GitHub project sites (https://github.com/ohdsi/).

**Ecosystem Collaboration:** The OHDSI consortium is organized by Projects and Workgroups, Annual Symposium and satellite events, and Community Forum [http://forums.ohdsi.org.](http://forums.ohdsi.org/)

**Security:** The technical leadership of the OHDSI collaborative holds regular discussions related to toolset security. Apache SHIRO is used for the securing the WebAPI component (https://www.ohdsi.org/web/wiki/doku.php?id=development:security).

**Legal:** Uses Open Source licenses such as Apache License 2.

**Finance:** OHDSI is an open collaborative that, as an organization, is not specifically funded by any single organization. However, numerous individuals and teams within the collaborative have received funds to focus on specific needs and topics. Funding sources have included private and public sources.

**Marketing:** The collaborative is not a business entity and so does not actively engage in marketing. There are regular discussions about disseminating knowledge and tools developed by the collaborative. There is also a specific workgroup focusing on dissemination (<https://www.ohdsi.org/web/wiki/doku.php?id=projects:workgroups:dissemination-wg>). Besides these mechanisms, researchers within the collaborative tend to provide acknowledgment to OHDSI in research publications and presentations (https://www.ohdsi.org/resources/publications/).

**Dependency Hygiene:** Depends on individual tools. The OHDSI WebAPI uses Maven for dependency management. The Methods library and several other projects, including patient level prediction, use R and R related toolsets such as RStudio.

## Use Case: R

(The R Project for Statistical Computing) <https://www.r-project.org/>

**Author of Use Case:** Davis M (U Pitt)

**Short History of Tool:** R is a language and environment for statistical computing and graphics. It is a GNU project similar to the S language and environment which was developed at Bell Laboratories (formerly AT&T, now Lucent Technologies) by John Chambers and colleagues. R can be considered as a different implementation of S. There are some important differences, but much code written for S runs unaltered under R. R provides a wide variety of statistical (linear and nonlinear modeling, classical statistical tests, time-series analysis, classification, clustering …) and graphical techniques, and is highly extensible. The S language is often the vehicle of choice for research in statistical methodology and R provides an Open Source route to participation in that activity. One of R’s strengths is the ease with which well-designed publication-quality plots can be produced, including mathematical symbols and formulae where needed. R is available as Free Software under the terms of the Free Software Foundation’s GNU General Public License in source code form. It compiles and runs on a wide variety of UNIX platforms and similar systems (including FreeBSD and Linux), Windows and MacOS.^1^

**Governance:** The current R is the result of a collaborative effort with contributions from all over the world. Since mid-1997 there has been an R Development Core Team responsible for overseeing its development, along with the R Foundation which is a not-for-profit organization working in the public interest. The R Foundation is seated in Vienna, Austria and currently hosted by the Vienna University of Economics and Business. It is a registered association under Austrian law and active worldwide. The Foundation continues to support the development of R and provides exploration of new methodologies, teaching and training of statistical computing and the organization of meetings and conferences with a statistical computing orientation. The ordinary members, of the Foundation, are elected by a majority vote of the general assembly. New ordinary members are selected based on their non-monetary contributions (code, effort …) to the R project.

**Documentation:** Various fully developed documentation is available for both statistical end-user, developer and administrators. R provides versions for the most recent released R version (R-release), a very current version for the patched release version (R-patched) and finally a version for the forthcoming R version that is still in development (R-devel). This documentation adequately covers a wide range of topics including guides for installation, writing R Extensions covers how to create your own packages, tutorials, a guide to the internal structures of R and coding standards for the core team working on R itself.

<https://cran.r-project.org/manuals.html>

**Code quality:** The R development core team (R Core) using the Apache Subversion as its software versioning and revision control system to maintain current and historical versions of files such as source code, web pages, and documentation. Reasonable software development and testing methodologies are employed by R Core in order to maximize the accuracy, reliability, and consistency of R’s performance. While some aspects of R’s development are handled collaboratively, others are handled by members of the team with specific interests and expertise in focused areas. R Core maintains guidelines for its Software Development Life Cycle (SDLC) https://www.r-project.org/doc/R-SDLC.pdf There have also been coding standards established providing guidance, https://cran.r-project.org/doc/manuals/R-ints.html

**Support:** R provides various support options from online self-help tools to primary FAQ listings which are periodically updated to reflect very commonly asked questions by R users. The R Project also maintains a number of subscription-based email lists for posing and answering questions about R, including the general R-help email list, the R-devel list for R code development, and R- package-devel list for developers of Comprehensive R Archive Network (CRAN) packages.

**Ecosystem collaboration:** The R Foundation actively supports two conference series, organized regularly by members from the R community:

- useR!, providing a forum to the R user community.
- DSC, a platform for developers of statistical software.

R Core does meet, collectively and/or in smaller groups, with a level of frequency dictated by multiple factors, including taking advantage of regularly scheduled conferences where members of R Core may already be in attendance. Such conferences include those that are specific to statistical computing and R itself (http://www.r-project.org/conferences.html). These routine communications and meetings ensure that the collaborative efforts are appropriately coordinated and prioritized as ongoing development takes place.

**Security:** The R project relies heavily on the development of functional software packages by community developers, which are submitted to a central repository, CRAN. Hence, there is an inherent security risk in malicious code making their way unnoticed into the central repositories. There are a few older published papers on the potential security risk of R and general guidelines on how to handle security issues.^2^ The CRAN does have some general guidelines for submitting new packages but doesn’t address any security regulations.

**Legal:** R is available as Free Software under the terms of the Free Software Foundation’s GNU General Public License in source code form. It compiles and runs on a wide variety of UNIX platforms and similar systems (including FreeBSD and Linux), Windows and MacOS.

**Finance:** R is supported largely by supporting members and “one-off donations” to the R Foundation. Any person or legal entity may become a supporting member of the R foundation by paying membership fees. Annual membership fees for supporting members are:

- Supporting natural persons: EUR 25
- Supporting institutions: EUR 250
- Supporting benefactors: EUR 500

**Marketing:** No direct information on marketing strategies could be located. However, R has gained market share by an “evangelist” approach amongst statisticians, data analysts and others from the biomedical community. R is comparable to popular commercial statistical packages, such as SAS, SPSS, and Stata, but R is available to users at no charge under a free software license. A number of commercial software vendors are providing commercial support and/or extensions to their products to support R extensions to their customers.

**Dependency Hygiene:** The R package itself is self-contained in general. Its “dependencies” rely on the contributions from its community of developer libraries within the central CRAN and its distribution under the terms of the Free Software Foundation’s GNU. Each package dependencies are clearly document within the package summary documentation within the CRAN site.

**References**

1. <https://www.r-project.org/>
2. [https://cran.r-](https://cran.r-project.org/web/packages/RAppArmor/vignettes/v55i07.pdf) [project.org/web/packages/RAppArmor/vignettes/v55i07.pdf](https://cran.r-project.org/web/packages/RAppArmor/vignettes/v55i07.pdf)

## Use Case: REDCap

https://www.project-redcap.org

<https://projectredcap.org/resources/community/>

https://projectredcap.org/software/

**Author of Use Case:** GQ Zhang

**Short History of the Tool:** REDCap (Research Electronic Data Capture) is a web-based electronic data capturing (EDC) software solution and workflow methodology, enabling data capture for clinical and translational research. REDCap uses institutional hosting as a “platform” to reach out and bring value to its intended end users.

REDCap was created in 2004 at Vanderbilt University. It was originally developed to support clinical researchers who needed a secure data collection tool that met HIPAA compliance standards. The REDCap consortium was officially launched in 2006. The consortium consists of non-profit organizations interested in expanding REDCap’s functionality through collaborative software development. Each partner site was given access to the codebase so that they could install their own REDCap system and offer it to their researchers. The REDCap annual in-person conferences were established in 2009, offering educational and networking opportunities to REDCap administrators around the world.

**Governance:** <https://github.com/vanderbilt-redcap> REDCap is not open-source software in the usual sense, as its code base is not open to an individual developer. REDCap is available at no charge to institutional partners and is restricted in use, permitted only for non-commercial research purposes. REDCap is also restricted in redistribution because Vanderbilt is the only entity that can distribute it. Any and all derived works – such as innovations or programmatic features added on by the user – are owned by Vanderbilt. [Wikipedia]

**Documentation:** Detailed documentation is available for set up and usage, but not on contributing source code. <https://projectredcap.org/resources/library/>

<https://projectredcap.org/about/>

<https://projectredcap.org/resources/community/>

**Code Quality:** REDCap source code is not open to the community. However, REDCap is used by 3372 Institutions in 130 Countries with 677k Projects and 926k Users. 6408 articles citing the use of REDCap.

**Support:** Contact [redcap@vanderbilt.edu](mailto:redcap@vanderbilt.edu) <https://projectredcap.org/partners/join/>

**Ecosystem Collaboration:** The REDCap Consortium has two tiers of users with distinct characteristics: institutional users and investigator users. Institutional users are responsible for installing, maintaining, and providing day-to-day support of the specific instance of the REDCap for their institution.

Investigator users - the ultimate end user, work within an instance of REDCap installed at an institution to create REDCap projects for their specific data capturing needs. Data capturing workflows are configurable according to specific project needs.

REDCap can be installed in a variety of environments for compliance with such standards as HIPAA, 21 CFR Part 11, FISMA (low, moderate, high), and international standards.

**Security:** The software application employs various methods to protect against malicious users who may attempt to identify and exploit any security vulnerabilities in the system. Input data or build an online survey or database from anywhere in the world over a secure web connection with authentication and data logging.

**Legal:** REDCap Non-Profit End-User License Agreement is made by and between Vanderbilt University (“Vanderbilt”), a not-for-profit corporation duly organized and existing under the laws of Tennessee and having offices at 1207 17th Avenue South, Suite 105, Nashville, Tennessee 37212.

**Finance:** Early support of REDCap was provided by NCRR.

**Dependency Hygiene** [Deployment environment requirement]**:** Web server with PHP (PHP 5.3.0+, including support for PHP 7). Apache (any OS) or Microsoft IIS (Windows).

MySQL database server (MySQL 5.0+, MariaDB 5.1+, or Percona Server 5.1+). a MySQL client (e.g., phpMyAdmin, MySQL Workbench) is required for performing installation/upgrades SMTP email server.
